# Precision Medicine for Cancer and Health Equity in Latin America: Generating Understanding for Policy and Health System Shaping

**DOI:** 10.3390/ijerph22081220

**Published:** 2025-08-05

**Authors:** Ana Rita González, Lizbeth Alexandra Acuña Merchán, Jorge A. Alatorre Alexander, Diego Kaen, Catalina Lopez-Correa, Claudio Martin, Allira Attwill, Teresa Marinetti, João Victor Rocha, Carlos Barrios

**Affiliations:** 1 Policy Wisdom LLC, Quebradillas 00678-2705, Puerto Rico; aattwill@policywisdom.com (A.A.); tmarinetti@policywisdom.com (T.M.); jrocha@policywisdom.com (J.V.R.); 2 Cuenta de Alto Costo, Cra. 45 #103 34 Oficina 802, Bogotá 110111, Colombia; l.acuna@cuentadealtocosto.org; 3 Health Pharma Professional Research S.A de C.V., Av. Insurgentes Sur 662-Piso 3, Col. Del Valle, Benito Juárez, Ciudad de México 03100, Mexico; jorge.alatorre@iner.gob.mx; 4 Centro Oncologico Riojano Integral, La Rioja F5300, Argentina; dlkaen@hotmail.com; 5 Genome Canada, 50 Metcalfe Street, Suite 2100, Ottawa, ON K2P 1P1, Canada; clopez@genomecanada.ca; 6 Alexander Fleming Institute, Av. Crámer 1180—C1426ANZ, Buenos Aires C1426, Argentina; cmartin@alexanderfleming.org; 7 LACOG—Latin American Cooperative Oncology Group, Porto Alegre 91900-580, Brazil; barrios@thummi.global

**Keywords:** precision medicine, biomarker testing, health equity, cancer care, cancer policies

## Abstract

This study presents and discusses evidence on the value of biomarker testing and precision medicine in Latin America through a health equity lens. It is essential to explore how to harness the benefits of precision medicine to narrow the health equity gap, ensuring all patients have access to the best cancer treatment. The methodology employed to develop this document consists of a non-systematic literature review, followed by a process of validation and feedback with a group of experts in relevant fields. Precision medicine could help reduce health inequities in Latin America by providing better diagnosis and treatment for everyone with cancer. However, its success in achieving this depends on the implementation of policies that promote equitable access. Findings indicate that the current policy landscape in the Latin American region is not conducive to improving access, reach, quality, or outcome-related problems in cancer care, nor to realizing the full potential of precision medicine. The study explores how precision medicine can advance health equity, concluding with an analysis of the challenges and recommendations for overcoming them.

## 1. Introduction

Medical science’s understanding of the molecular basis of diseases has made remarkable advancements, leading to the development of innovative, effective, and targeted treatments. However, these advancements have brought to the forefront issues related to equity, access, affordability, and the allocation of precision medicine resources. It has created new tensions in the old debate of “who gets, who pays, and who gets paid” in healthcare.

This issue is particularly pronounced in the field of oncology, given that cancer is fundamentally a genetic disease, with genomic technologies and targeted drug development radically transforming its treatment landscape. Despite the potential of targeted therapies to effectively inhibit cancer growth with minimal harm to normal cells, challenges exist, such as the development of treatment resistance in cancer cells and the difficulty in creating treatments for certain targets [1].

Precision medicine is defined as an emerging approach to disease treatment and prevention that considers individual variability in genes, environment, and lifestyle [2]. Biomarker testing is a laboratory method that uses a sample of tissue, blood, or other body fluid to check for certain genes, proteins, or other molecules that may be a sign of a disease or condition, including cancer. Biomarker testing can also be used to check for certain changes in a gene or chromosome that may increase a person’s risk of developing cancer or other diseases [3]. Biomarker testing can support the development of targeted therapy, which is a type of cancer treatment that targets proteins that control how cancer cells grow, divide, and spread [4]. Therefore, in the context of cancer, precision medicine involves analyzing the genetic mutations and specific characteristics of a patient’s tumor to design targeted therapies, relying on the use of biomarker testing. It utilizes a person’s genetic profile to guide disease prevention, diagnosis, and treatment decisions. By analyzing genetic mutations and specific tumor characteristics, precision medicine tailors treatments for patients, enhancing treatment effectiveness and reducing avoidable side effects, time, and costs involved in trial-and-error approaches [5,6].

While precision medicine has revolutionized cancer care, socioeconomic disparities remain a significant concern, given that social and economic factors strongly influence health outcomes and access to care. Across all income levels, health follows a social gradient; those in lower socioeconomic positions consistently experience worse health outcomes and more limited access to essential services, undermining equity in cancer care [7]. Achieving health equity, which aims to eliminate avoidable differences in health among different social, economic, and demographic groups, is imperative [8].

Ensuring equitable access to precision medicine is critical to reducing broader health inequities and providing comprehensive cancer care for all [9,10]. In Latin America (LatAm), cancer has a substantial impact and is one of the leading causes of morbidity and mortality. The cancer incidence in LatAm is generally lower than in developed countries, but the mortality is significantly higher. The cancer mortality-to-incidence ratio in 2022 for LatAm is 0.50, compared to 0.25 in the US [11,12], indicating worse cancer outcomes in the region. Patients are less likely to develop cancer but more likely to die from it when they do. This is partly due to diagnosis at later stages of the disease, as well as barriers to accessing the health system or the low quality of health systems in LatAm [13]. Projections of the year 2030 show that cancer cases will increase by 35% in South America overall and 42% in Mexico [14]. This situation demonstrates an enormous need for, and the significant value of, precision medicine in the region.

There are numerous challenges to effective healthcare in LatAm, including cancer care, which contribute to health inequities. These include but are not limited to barriers to accessing early detection and screening programs, insufficient healthcare infrastructure, a shortage of specialized healthcare professionals, suboptimal quality of health services, and inequities in the availability of advanced treatments.

In essence, precision medicine has the potential to reduce health inequities in LatAm by improving health outcomes for all people living with cancer through accurate diagnosis and targeted treatment. However, for it to do so, it must first be implemented in health systems and applied in a way that is intentionally equitable.

This review provides a summary of evidence for subject matter experts to discuss and refer to in reaching a consensus on the importance and value of precision medicine for cancer care in the LatAm region. This is an essential landmark in this emerging field because there has been a lack of expert consensus on this topic, a prerequisite for driving patient access to the best available cancer treatment options and improving health equity in LatAm. The broader consensus-building process this document facilitates seeks to discuss, understand, and agree on how best to harness the benefits of precision medicine to narrow the health equity gap in LatAm.

## 2. Objectives and Methodologies

The objective of this consensus document is to present and discuss evidence and position the importance and value of biomarker testing and precision medicine in LatAm through a health equity lens. It seeks to encourage dialogue on the best ways to advance broad and deep patient access to the best cancer treatment options, with a special focus on Argentina, Brazil, Colombia, Mexico, and Panama.

The methodology employed to develop this document consists of a non-systematic literature review, followed by a process of validation and feedback with a group of experts in relevant fields.

We collected and analyzed evidence at three levels: global, which includes studies, data from around the world, and conceptual evidence not linked to a specific geography; regional, encompassing research and reports specific to LatAm; and country-level, with information focused specifically on five focus countries: Argentina, Brazil, Colombia, Mexico, and Panama. The search was conducted on peer-reviewed literature, policies, and policy recommendations by national and international organizations, on the topics of (a) the current position of cancer care in LatAm, (b) current policy landscape and adoption of precision medicine, (c) precision medicine and cancer, (d) precision medicine, benefits, and health equity. These topics were purposively selected as part of a gap assessment approach commonly used to inform action-oriented strategies. They offer a structured view of the current state and future potential of precision medicine in cancer care, along with the relevant policy and health system context—providing a foundation to identify challenges and formulate recommendations for advancing equitable precision medicine in the region.

A research template for these four sections was built, and two researchers fluent in English, Spanish, and Portuguese searched for peer-reviewed studies in these languages that provide evidence to answer the proposed questions. Peer-reviewed studies had to report on data from the last ten years, unless they were discussing terms and concepts. The search was conducted on BioMed Central, PubMed, and Google Scholar between 26 February and 9 April 2024. A total of 133 papers were considered in the preliminary literature review. These studies were selected through a targeted search covering the four topics or dimensions mentioned above and prioritizing those that provided relevant empirical data or policy analysis. Studies were excluded if they were outside the timeframe (unless conceptual), unrelated to the core topics, focused solely on clinical or biological mechanisms without policy or equity relevance, or lacking sufficient methodological detail. Additionally, the search was conducted on official governmental, national, and international websites to identify policy documents and reports on the policy landscape.

Experts from LatAm were selected based on expertise and academic merit, which was assessed based on the content and quantity of relevant publications in the subject field, affiliations, linkages to relevant medical societies, evidence of engagement and participation in decision-making processes of cancer policy or guidelines, and records showing that the expert is up to date with oncology care and precision medicine. Their disciplinary backgrounds include oncology, surgery, internal medicine, biosciences, and human genetics.

The findings from the literature review were collected, synthesized, and appraised to ensure relevance to the topics and then organized into a working document covering the key topics of cancer and health equity in LatAm, the role of precision medicine, the policy landscape, existing challenges, and recommendations to address identified gaps. These findings were discussed and validated by seven LatAm experts during online panel sessions held on 29 October and 12 November 2024, followed by several rounds of offline review and calls. This manuscript was developed in parallel with the expert feedback process and integrates key insights from both the literature and expert contributions. The seven experts who participated throughout the process are listed as co-authors.

## 3. Background

Cancer is one of the leading causes of death in LatAm and the Caribbean (LAC) [15], constituting an important public health issue and associated with high societal and economic costs. It is estimated that between 30 and 50% of cancer deaths could be prevented by modifying or avoiding key risk factors and implementing existing evidence-based prevention strategies [16]. Furthermore, it is estimated that the cancer burden in LAC will increase by 67%, reaching 2.4 million new cases annually by 2040 [17]. This is the result of rapid growth, population ageing, and lifestyle and environmental factors [13]. These figures highlight the importance of developing more stringent and targeted cancer care. Progress in improving care increasingly relies upon the use of precision medicine, in which biomarker testing is conducted to guide clinical decisions that can lead to better health outcomes and quality of life for people living with cancer.

Socioeconomic inequalities exist across the cancer continuum, including exposure to/prevention of risk factors, access to effective cancer prevention interventions, and early detection, diagnosis, treatment, and palliative care [18,19]. Health equity for cancer is especially relevant for LatAm as this is a diverse region regarding socioeconomic, demographic, and cultural conditions [19], with one in three people living in poverty [20]. Data indicates that the percentage of the population living below the national poverty line was notably high in Argentina (39.2%), Colombia (36.6%), Mexico (36.2%), and Brazil (31.6%) in 2022. Panama had a significantly lower rate of 21.8% in 2021 [21,22].

While precision medicine and the use of omics or genomic biomarkers can potentially improve cancer health outcomes, they are also subject to issues of health equity in access and implementation, and these tools will either widen or narrow existing inequities [10].

### 3.1. Impact of Cancer in LatAm and the Caribbean

Globally, nearly 20 million new cases of cancer were reported in 2022 and accounted for nearly 10% of the world’s total disability-adjusted life years (DALYs) registered in 2019 [12,23]. Approximately 1.5 million of these cases were reported in LAC, representing 7.7% of new cases registered worldwide [12,24,25]. Figures for LatAm would be only slightly lower, as more than 90% of the LAC population resides in LatAm [25]. Almost 10 million cancer-related deaths were registered in 2022 globally [12], making cancer a leading cause of death, and around 750,000 of those deaths were registered in LAC [24]. Considering that 30 to 50% of all cancer cases are preventable [16], between 450,000 to 750,000 cases could have been avoided in LAC in 2022 through lifestyle changes and proactive health measures. The annual number of cancer cases in Latin America and the Caribbean is estimated to increase by 85.1% between 2022 and 2050, rising from 1.5 million to 2.9 million cases [26], highlighting the urgent need for improving care in the region.

The incidence and mortality rates underscore the significance of cancer as a pressing public health concern globally and in LatAm, requiring comprehensive strategies for prevention, early detection, and adequate treatment. Regarding absolute numbers, Brazil had the highest cancer incidence in 2022 among the focus countries, followed by Mexico, Argentina, Colombia, and Panama. Based on age-standardized cancer incidence and mortality rates, Argentina and Brazil are disproportionally affected compared to Colombia, Mexico, and Panama. Nonetheless, in all these countries, cancer represents between 8.47 (in Mexico) and 15.76% (in Argentina) of total Disability-Adjusted Life Years (DALYs) registered in 2019 [23]. The most frequent cancers worldwide and in LAC in 2022 were lung, breast, colorectal, prostate, and stomach [12,24]. Table 1 presents the impact of cancer globally, regionally, and in the focus countries [12,23,24,27,28,29,30,31]. It is likely that these values underestimate the true burden of cancer in the region. The quality of national estimates depends on factors such as the coverage, accuracy, and timeliness of recorded incidence and mortality data [17]. Furthermore, not all countries in the region have population-based cancer registries, and those that do exhibit varying levels of quality and population coverage [32]. Finally, patients may not have access to facilities capable of confirming cancer diagnoses, and this underdiagnosis contributes to underestimation of the actual data.

The increasing cancer burden exerts significant strain on populations and health systems at all income levels globally. The high costs of diagnosis and treatment, the need for specialized medical professionals and infrastructure, and long-term care represent important challenges, while the current budgetary allocation and global resource mobilization are markedly insufficient [33]. It is important to note the significant economic differences among these countries, particularly when comparing their nominal income levels (in USD) to those adjusted for purchasing power parity (PPP), which accounts for cost of living. In 2023, GDP PPP per capita and nominal GDP per capita were as follows: Argentina: USD 30,082 vs. USD 14,187; Brazil: USD 21,107 vs. USD 10,295; Colombia: USD 20,676 vs. USD 6947; Mexico: USD 24,767 vs. USD 13,790; and Panama: USD 39,803 vs. USD 18,686 [34]. Another challenge is the rising incidence of cancer among adolescents and young adults since the 1990s. The underlying causes remain largely unassessed and poorly understood [35,36], yet this trend has significant implications for screening, diagnosis, treatment, and management, and risks exacerbating the strain on health systems and society.

Cancer has deeply negative socioeconomic and psychosocial implications, manifesting as direct and indirect costs and impact on life course, labor market disadvantage, social support, physical and mental health, and social vulnerability [37,38]. A study from 2023 estimated that cancer will cost the global economy 25.2 trillion international dollars (INT$) between 2020 and 2050, which is equivalent to 0.55% of the global GDP [39]. The same study estimated total macroeconomic costs in 2017 INT $, using a 2% discount rate. In LAC, costs totalled 960 billion INT$, with direct costs to health systems accounting for approximately 12% of total costs, and lost productivity according to the human capital approach accounting for the other 88% [39]. For the focus countries, cancer costs for the period 2020 to 2050 in INT$ were estimated at 106 million in Argentina, 192 million in Brazil, 91 million in Colombia, 172 million in Mexico, and 16 million in Panama [39].

Cancer has a significant negative impact on the well-being of individuals and caregivers, including on their mental health and personal and work relationships. Quality of life, referring to the whole, integrated state of physical, mental, and socioemotional well-being, is an important outcome criterion in oncology [40]. Facing cancer, the wide variety of side effects that can arise, and possible sequelae can lead to significant health-related complaints and psychological distress [40]. The side effects from cancer treatments make it even more important to ensure the right patient gets the right treatment at the right time. A study in Colombia found that breast cancer patients reported worse quality of life than the general population regarding role, emotional, cognitive, and social functioning scales [41]. Another study in this country also found worse quality of life among cancer patients than the general population, including experiencing financial difficulties [42].

A systematic review in Brazil found that the quality of life of patients diagnosed with cancer in the country is negatively impacted by pain, fatigue, sleep disturbances, and depression. Patients who have medical procedures or devices such as ostomy, mastectomy, or colostomy bags report even worse quality of life [43].

Evidence from other LatAm countries highlights the impact that different types of cancer have on patient quality of life. For breast cancer, a study in Colombia found that factors associated with low quality of life were mammary symptoms, side effects of systemic treatment, decreased sexual pleasure, and reduced future expectations [44]. The stage of the disease can have a significant impact on patients, as a systematic review and meta-analysis of breast cancer patients in LAC found that those with metastatic disease reported lower quality of life and higher symptom burden compared to patients at earlier stages [45]. Mexican prostate cancer patients showed a negative impact on quality of life after radical prostatectomy surgery, related to incontinence and erectile dysfunction [46], while colorectal cancer patients reported distress and post-traumatic stress [47]. Cancer exerts tremendous physical, emotional, and financial stress on individuals, families, communities, and healthcare systems globally [16]. Therefore, as the cancer burden continues to grow, it is necessary to promote prevention, early diagnosis, high-quality treatment, and adequate survivorship care. This indicates the importance of early and accurate diagnosis and more targeted, more personalized, less intrusive treatment. Therefore, precision medicine has the potential to avert as much suffering and cost as possible.

Cancer affects not only patients, but also their families and caregivers, who face emotional, social, physical, and financial problems that affect their quality of life [48]. This is especially concerning in resource-constrained countries, where the formal care system cannot cater to all the needs of all cancer patients, leading to family members, friends, or neighbors of patients engaging in informal caregiving [49]. Informal caregivers are the main source of care provision for people in a situation of dependency in LatAm [50]. Informal carers are often challenged to reconcile their work responsibilities and other responsibilities in their personal lives with providing care [51], and they may have to reduce their working hours or even quit their jobs to meet care responsibilities [52], which can lead to increased social and economic inequality.

### 3.2. Challenges for Cancer Care in LatAm

The significant impact cancer has on epidemiological, socioeconomic, and well-being dimensions highlights the importance of improving access to optimal care. Cancer control requires a comprehensive approach regarding prevention, screening and early detection, fast treatment of new cancer cases, follow-up survivorship, and palliative and end-of-life care, each of which requires unique activities and supporting policies [53]. There are important challenges in LatAm that hinder the provision of cancer care, with studies published in 2021 and 2023 discussing some of these challenges, such as delayed diagnosis or treatment initiation, lack of access to high-cost drugs, insufficient investment in cancer control, and nonexistent, inadequate, siloed, or inaccessible cancer registries [32,54].

Delays in cancer diagnosis and treatment contribute to the substantial burden it represents in LatAm [55,56], with studies indicating that delays are related to financial, geographic, and organizational barriers to access, such as fragmented healthcare systems, poor cancer suspicion in primary healthcare (where patients spend considerable time and there is limited access to advanced diagnostic tools), and insufficient/poor coordination among different levels of care and healthcare providers [57,58]. The time spent on bureaucracy, securing specialist appointments, and diagnostic tests results in late-stage cancer presentations, lowering the chances of effective treatment. Other aspects, such as cultural barriers (fear of suffering, dying, and abandoning the family), stigma for some types of cancers (particularly reproductive cancers), misconceptions about the in/curability of cancer, and low health literacy and cancer awareness, further contribute to delays in detection and treatment [32]. Patients in LatAm mostly receive healthcare through public health systems, which, compared to private healthcare in the region, have lower rates of cancer screening, more delays in cancer diagnosis, and greater proportions of advanced disease at presentation [59].

In fact, there are vast differences in access to and quality of cancer care across LatAm that result mainly from insurance types [60]. This is evidenced by studies from Brazil and Mexico, which show that patients with breast or lung cancer and public healthcare coverage were diagnosed more frequently at advanced clinical stage than privately insured patients [61,62,63]. A systematic review in LatAm also found that being covered by health insurance was a major determinant in participating in cancer screening [64]. In addition, most precision medicine in LatAm is delivered within the private component of local healthcare systems, as public healthcare institutions are not financially capable of covering these advanced testing techniques and therapies [65]. Therefore, innovative anticancer drugs are also often only accessible to those enrolled in private insurance programs [14], as evidenced by a study in Mexico that found that eight innovative essential cancer medicines were covered by social health insurance, but only three were covered in the public sector [66].

High-cost drugs are often inaccessible in LatAm countries. A study from 2017 found that of 37 new cancer drugs launched worldwide between 2009 and 2013, only 17 were available in Mexico and 10 in Brazil [14]. The availability of drugs influences oncologists’ decisions when prescribing targeted therapies [65]. When citizens are unable to access therapies through the regular health system channels, they may resort to filing lawsuits against the government, given that access to health is recognized as a constitutional right in most LatAm countries. Such judicial processes are frequently used by patients to access high-cost therapies in Colombia and Brazil [14]. A study from 2019 discussed that the overwhelming number of healthcare-access-related lawsuits in Colombia has made litigation an inefficient means of obtaining access to medicines, and it even delays patients’ access to healthcare [67]. In Brazil, a study from 2020 discussed that judicialization creates costly, unscheduled expenses, displacing budget for other sectors of the Ministry of Health, such as the supply of drugs to primary care and the treatment of patients with sexually transmitted infections and AIDS [68]. Despite such negative impacts, studies discuss that the reduction in the costs of medicines from lawsuits can benefit society [68], with judicialization representing an important mechanism for state accountability [69]. In fact, a 2023 study in Brazil found that litigation was significantly associated with improved cancer survival rates, comparing groups that accessed certain drugs or treatments through the court system with those that did not [70]. The discussion on the judicialization of health is complex, and its impact on health equity is inconclusive [69,71].

Regarding investment in healthcare, considering the heterogeneity in the region and the few comparative studies on the cost of cancer, per capita spending can provide some illustrative information on the topic [32]. In 2018, the average total healthcare spending per capita was USD 666 in LAC, compared to USD 4315 in the United Kingdom [32]. In terms of cancer-specific spending in countries in LAC, on average, 0.12% of gross per-capita national income was spent on cancer, compared to 0.51% in the UK, 0.60% in Japan, and 1.02% in the USA [32]. The differences in investment in healthcare are also noted when comparing country-level current health expenditure as a percentage of GDP between LatAm and high-income countries. The WHO recommends that governments spend at least 5% of their GDP on health [72]. In 2019, this value was 5.95% in Argentina, 6.28% in Colombia, 3.92% in Brazil, and 2.68% in Mexico, while it was above 7 in Canada, Australia, and New Zealand, and above 9 in Germany, France, the US, and Japan [73].

Suboptimal cancer registries significantly hamper cancer surveillance and research in LatAm, and subsequently, cancer policy planning and responses. In fact, less than 3% of the population of Central America and 10% of that of South America are covered by high-quality cancer registries [74]. A study published in 2021 discussed that in 2020, population-based cancer registries covered 41% of the population in Argentina, 22% in Brazil, 13% in Mexico, and 100% in Panama. However, the quality of registries regarding comparability, completeness, validity, and timeliness is very low [32].

### 3.3. Health Equity and Cancer

Health equity, defined by the World Health Organization (WHO) in the early 1990s as “the absence of unfair, avoidable or remediable differences in health among population groups, defined by social, economic, demographic or geographic characteristics” [75], has long been a primary goal of public health. Therefore, healthcare is equitable when resource allocation and access are determined by health needs [76] and when everyone has a fair opportunity to attain their full health potential [77].

The terms *inequality* and *inequity* are sometimes used interchangeably [78], but there are important differences between them. Inequality refers to any differences between individuals or population groups, so it can refer to any measurable aspect of health that varies across individuals or groups. Inequity is an ethical value and refers to differences that are unnecessary, avoidable, unfair, or unjust. The key distinction between inequality and inequity is that inequality simply describes differences, while inequity implies that these differences are unnecessary, avoidable, and morally wrong. Therefore, not all inequalities are unjust, but all inequities in healthcare stem from unjust inequalities [79,80]. A difference in health outcomes between younger and older adults due to age-related factors, for example, is an inequality because it reflects natural variations. However, a disparity in access to healthcare between wealthy and poor communities, leading to worse outcomes for the latter, is an inequity because it is unnecessary, avoidable, and rooted in social injustice.

The origins of this research can be traced back to the early 1800s, originating from the disciplines of sociology and political economics, and is embedded in the development of public health [81]. In the 1940s, the WHO and the United Nations General Assembly declared that every human being is entitled to the highest attainable standard of health, regardless of race, religion, political belief, or socioeconomic status, establishing fundamental principles for health equity [82,83].

Since the late 1970s, equity in the context of health has become a central objective for the WHO, as attributed in the Alma-Ata Declaration of 1978, which emphasized the unacceptable nature of global health inequities [84]. Research on health equity entered a period of rapid development between 1991 and 2005, bolstered by the official endorsement of the “concept and principles of equity in health” by the WHO in the early 1990s [77]. Building on this foundation, the WHO Commission on Social Determinants of Health was formed in 2005 to provide a systematic framework for health equity studies [7].

Health equity was a cornerstone of the Millennium Development Goals and a major milestone in the Sustainable Development Goals [85]. Today, the value of health equity is understood to address and resolve inequalities in health outcomes, ensuring that everyone has the opportunity to achieve their highest possible standard of health.

Robust evidence demonstrates that opportunities to be healthy depend on social, economic, demographic, geographic, and other factors and dimensions of inequality that vary across groups [8,86]. Even in the US, where significant resources are invested in health and advancements in precision medicine are evident, significant inequities persist, particularly among vulnerable populations such as Hispanic and Black communities [87,88], especially when it comes to the use of new technologies such as genomics, genetic testing, and biomarker analysis for precision health [10]. Access alone does not guarantee equity; coverage must be equitable, and this remains one of the main challenges for health systems in the region. Furthermore, there must be robust data capable of measuring trends and access to ensure that equity is not only pursued but also effective. The features and structures of healthcare systems significantly influence inequities, shaping how care is accessed and delivered. Fragmented healthcare systems, such as those in LatAm countries, often exacerbate these inequities, a reality that is frequently overlooked by decision-makers. Therefore, achieving health equity requires intentional effort from a healthcare perspective, alongside broader initiatives that tackle poverty, discrimination, lack of social power and participation, and insufficient access to a range of resources, services, and conditions needed for optimal health [86].

Empirical evidence suggests that public policy plays a central role in addressing sources of inequity, as illustrated by targeted actions improving inequalities in access and use of health services in the Americas [89]. A study from 2021 analyzed 32 national health sector policies, strategies, and plans from LAC to understand how equity is being addressed in the region, with findings indicating that almost all countries included health equity as part of their health plan’s mission or vision, as well as addressing underlying determinants of health [89].

Such findings provide an important opportunity to advance discussions regarding health equity in the region. This is necessary, as the region’s health systems are deeply fragmented and segmented, hindering the provision of quality care and overall equity levels in health and society [90].

The WHO lists the following evidence-informed actions that are needed to improve health equity [8]:

1. The health sector needs to ensure that high-quality and effective services are available, accessible, and acceptable to everyone, everywhere, when they need them.
2. Health and other sectors need to act on the wider structural determinants of health to tackle the inequitable distribution of power and resources, and to improve daily living conditions.
3. The health sector needs to take the lead in monitoring health inequities through monitoring health outcomes and health service delivery, as well as working with other sectors to monitor people’s living conditions.

The WHO also offers key examples of policies and frameworks that are supportive of equitable healthcare. These include the following [8]:

- Redesigning health systems for equity, e.g., pooling financial resources to enhance redistributive capacity.
- Prioritizing the primary health care approach, e.g., investment of 1% of GDP in PHC.
- Tackling structural determinants such as sexism, racism, ageism, classism, and ableism.
- Addressing harmful gender norms and gender inequalities in health policies/services/programs, and having more women in leadership positions and decision-making processes.
- Protecting and increasing investment in health and other social sectors (through universal health coverage [UHC], education, and broader social protection).
- Ensuring equitable services and infrastructure in both urban and rural areas to ensure everyone can lead healthy lives.
- Continuing to monitor health inequalities and the impact of action.

The US Centers for Disease Control and Prevention (CDC) defines equity in cancer care as a scenario where everyone has an equal opportunity to prevent cancer, find it early, and get proper treatment and follow-up after treatment is completed [91]. Cancer inequalities refer to the unequal burden of cancer incidence, prevalence, mortality, survivorship, financial burden, screening rates, and stage at diagnosis among different population groups due to various factors, including socioeconomic status, race/ethnicity, geographic location, and access to healthcare [92,93].

Specific barriers to accessing healthcare include economic, geographic, epidemiological, and cultural factors [94]. High out-of-pocket costs for healthcare services can prevent individuals from accessing care; those in remote or rural areas may not have sufficient healthcare facilities or professionals available nearby, and they may fail to reach secondary or tertiary care facilities capable of confirming cancer diagnoses; outbreaks and prevalence of diseases can strain healthcare systems, limiting their capacity to provide adequate care to all individuals; cultural factors can influence healthcare-seeking behavior and healthcare provider–patient relationship; and weak health systems, including insufficient infrastructure, inefficient referral systems, a lack of healthcare workers, and inadequate supply chains, can impede the delivery of effective healthcare services [15,95,96,97,98,99,100].

In LatAm, UHC is still lacking [101], despite it being an important policy agenda for many countries, including those analyzed in this study [102]. Achieving universal coverage alone does not ensure equitable access, as systemic barriers and social determinants of health often hinder certain populations from fully benefiting from available services. In fact, the concept of universal coverage can sometimes conflict with the goal of equity. While universal coverage aims to provide access to healthcare for everyone, equity focuses on meeting individuals’ specific needs and ensuring they are involved in decision-making processes. Although PAHO’s definition of universal health coverage incorporates equity—stating that “Universal health is not just about ensuring everyone is covered, but that everyone has access to care when they need it, wherever they are” [103]—not all countries in the region that have advanced toward UHC have sufficient evidence to evaluate whether equitable access to healthcare has been achieved in practice [102]. Healthcare services in the focus countries are generally accessible and affordable, but there are still challenges in fulfilling the population’s health needs and managing the increasing costs of healthcare [102]. Although cancer care is covered by almost all public services, high-cost drugs are less commonly included in the public system’s bundle of services and, therefore, often inaccessible [54].

In Argentina [104], Brazil [105], Colombia [106], Mexico [107], and Panama [108], citizens have a constitutional right to health. The state must provide comprehensive, universal, and equal access to the healthcare system by law, which may trigger judicial cases regarding healthcare, where a lawsuit is filed against the government to obtain access to therapies. This process can increase inequity in drug access, as only those who can afford to bear legal costs pursue this avenue [109]. Overall, the provision of cancer care in health systems in LatAm faces major limitations regarding access to trained healthcare professionals and new therapies and adequate facilities for cancer care, due to inadequately distributed budgets across locations and geographical and cultural barriers [110]. Geographic and service-related factors, such as travel distances and waiting times, often play a determining role in timely healthcare access [111] and represent significant challenges in LatAm. In Brazil, for example, more than half of cancer patients are required to travel to and from their hometowns to another city to receive treatment [112], and similar geographic barriers, such as availability and cost of transportation, quality of roads, and long travel times, have also been reported in studies in Argentina, Colombia, and Mexico [113,114].

Health systems should promote access to socially, culturally, and linguistically appropriate, respectful, and high-quality cancer care [115], with the potential of reducing inequalities in the occurrence of cancer and advancing the cause of health equity [116]. Promoting equitable cancer care is necessary to ensure patients can access timely and adequate prevention and diagnostic services and receive optimal care, thus achieving equitable health outcomes. This study aims to highlight the importance and value of promoting biomarker testing and precision medicine in LatAm while emphasizing the need to ensure health equity.

## 4. Literature Review

This section presents the literature review findings and is divided into Section 4.1 and Section 4.2. Section 4.1 provides an overview of the literature on precision medicine, focusing on its application in cancer care through the utilization of biomarkers, discussing both current advancements and future prospects for enhancing diagnosis and treatment efficacy. It is divided into sections on precision medicine and oncology; the value of precision medicine; the policy landscape of precision medicine and oncology in LatAm; and the value of precision medicine for health equity. Section 4.2 presents information on challenges regarding the scientific development of precision medicine; challenges implementing precision medicine in healthcare delivery services; economic issues; and challenges relating to policies and regulations.

### 4.1. Literature Review of Precision Medicine, Its Application, Advancements, and Future Prospects

#### 4.1.1. Precision Medicine

The US National Human Genome Research Institute offers the following definition: “Precision medicine (generally considered analogous to personalized medicine or individualized medicine) is an innovative approach that uses information about an individual’s genomic, environmental, and lifestyle information to guide decisions related to their medical management. The goal of precision medicine is to provide a more precise approach for the prevention, diagnosis, and treatment of disease” [117].

Medicine has had a personalized approach since its nascence, with treatments tailored to individuals’ unique needs [118,119,120]. In fact, the fundamentals of precision medicine can be traced back to the ancient Greek era, when it was widely accepted that medicine cannot be generalized for everybody and the same treatment may not be suitable for everybody, given that each body/organism is different. Thus, appropriate treatment should consider patients’ individual characteristics, such as different health statuses and lifestyles [121].

It is vital to note that while precision medicine is inherently patient-focused, its application is not automatically patient-centered. Patient-centered care entails delivering clinical services that incorporate individual patient preferences, concerns, and needs, and ensures that patient values inform all treatment decisions. The goal of patient-centered care is to empower patients to be informed decision-makers by providing whole-person care that is both compassionate and empathetic [122]. A 2021 study offers a framework that shapes goals for precision medicine-based approaches to reflect patient values and shared decision-making that is continuously refined by utilizing a population-based evidence assessment repository to achieve personalized care [123]. The authors argue that they see such an approach (which is gradual, evidence-based, and patient-focused) as the progression of precision medicine. The proposed “PEAR” (population-based evidence assessment repository) framework involves customizing treatment based on tissue or receptor types and considering patients’ preferences, characteristics, and overall health. Authors acknowledge that, although initial training and infrastructure are necessary, the benefits in terms of better outcomes, compassionate care, patient involvement, and utilization of rapidly expanding population-based data would outweigh the initial investment. Furthermore, it would help drive precision medicine’s evolution to tailor therapeutic treatment beyond tissue or receptor types to therapy based on the patient’s preferences, characteristics, and complete health state [123].

The scientific advancements in genomics, such as the completion of the Human Genome Project in 2003 [124], and the development of next-generation sequencing that allowed a dramatic decrease in the cost of sequencing and genomic testing [125], fostered the development of precision medicine, as genetic markers associated with various diseases can support prompt and accurate diagnosis, risk stratification based upon genotype, and the capacity for tailored treatments [126]. Precision medicine became more widely known with the “Precision Medicine Initiative,” launched in the US in 2015, aiming to personalize medical treatment based on neurobiological differentiation. This initiative had a budget of USD 215 million for a million-person national research cohort, including public and private partnerships with academic medical centers, researchers, foundations, privacy experts, medical ethicists, and medical product innovators [127].

Today, the concept of precision medicine is used in a broader sense, describing an innovative approach to tailoring disease prevention and treatment, considering differences in people’s genes, environments, and lifestyles [117,128].

Precision medicine is incorporated into clinical practice through various tools, such as omics (the study of various aspects of a biological system, with genomics, proteomics, and transcriptomics being big data), artificial intelligence (AI), and machine learning, as well as the integration of environmental, social, and behavioral factors [129].

Genomics is the study of the complete set of DNA, including all its genes, in a person or other organism [130]. Some omics technologies include the following [131]:

- Epigenomics: Chemical modifications of DNA, histones, non-histone chromatin proteins, and nuclear RNA;
- Transcriptomics: Gene expression pattern in a cell/tissue;
- Proteomics: Proteins expressed by a biological system;
- Metabolomics: Metabolites and their fluctuations related to internal (genetic) and external factors (environment);
- Phenomics: Measurable physical and chemical outcomes of the interactions between genes and the environment that are experienced by individuals and influence their phenotypes.

Behavioral and social factors, such as diet, exercise, mental health, smoking status, and social support, as well as environmental factors, can influence health and disease and are important components of precision medicine [132]. Genome-wide association studies have found that the contribution of specific genes to some complex diseases, including cancer, may be very small, highlighting the need to integrate genetic and epigenetic effects with environmental, social, and behavioral data [133].

Essential components are needed for the incorporation of precision medicine into clinical practice, namely data, tools and systems, regulation, and key stakeholders [134].

Due to the vast volume and complexity of data used and required for precision medicine approaches, big data, AI, and machine learning play crucial roles in advancing the field. They are needed for data integration and analysis, which are crucial to the development of predictive models of disease progression, treatment response, and health outcomes, in addition to supporting clinical decision-making [135,136,137]. A massive amount of multifaceted data is needed to direct the development of new targeted drugs and approaches as envisaged by precision medicine [138]. Therefore, large-scale, detailed, and highly integrated tools and systems are required to collect, manage, and analyze these complex patient datasets. A clear regulatory structure is required to preserve privacy and ensure adherence to ethics and safety in precision medicine, in addition to fostering patient/provider trust and transparency around how patient data is stored, secured, protected, and/or shared [134].

It is important that medical professionals have the necessary expertise and education to prescribe the test and to correctly interpret the genomics profiles of their patients to implement precision medicine into clinical practice. This may require medical curriculum reforms and training in data analysis and interpretation of precision model outputs [134,139]. Academic and research universities, public and private healthcare institutions, industries, and governments must collaborate and play an active role in supporting the construction of a precision medicine policy framework [140,141]. In LatAm, current regulatory frameworks may not promptly provide patients with innovative drugs to fulfil their unmet medical needs, due to either specific prohibitive regulations or a lack of flexibility in the regulations [142]. Frameworks must facilitate quicker and safer access of patients to new drugs and foster more effective drug development by improving scientific resource utilization and enhancing patients’ participation [142].

The adjustment of treatments to individual or subgroups of patients is based on the use of disease-specific biomarkers [143]. Biomarkers are defined by the WHO as “almost any measurement reflecting an interaction between a biological system and a potential hazard, which may be chemical, physical, or biological. The measured response may be functional and physiological, biochemical at the cellular level, or a molecular interaction” [144]. The Biomarkers, EndpointS and other Tools (BEST) glossary defines a biomarker as a defined characteristic that is measured as an indicator of normal biological processes, pathogenic processes, or responses to an exposure or intervention, including therapeutic interventions [145]. BEST defines seven biomarker categories: susceptibility/risk, diagnostic, monitoring, prognostic, predictive, pharmacodynamic/response, and safety.

Biomarkers may be diagnostic, prognostic, or predictive. A diagnostic biomarker detects or confirms the presence of a disease or condition of interest or identifies an individual with a subtype of the disease [146]. A prognostic biomarker serves to predict disease aggressiveness, reflecting patient prognosis and survival. A predictive biomarker differentiates patients who are likely to benefit from a particular treatment and patients who are not, indicating whether a particular treatment brings clinical benefits to patients [147,148].

#### 4.1.2. Precision Medicine and Oncology

Cancer etiology depends on complex interplays at the genomic, transcriptional, proteomic, and metabolic levels; therefore, an in-depth investigation of tumors at the omics level can help understand the complex nature of tumor biology, including tumor evolution, heterogeneity, microenvironment, immune evasion, and drug resistance. Cancer may be influenced by a combination of genetics, lifestyle, social behavior, and environmental determinants of health. The CDC has recognized the importance of integrating both social/behavioral and genomic sciences to effectively conduct precision medicine for cancer.

Omics technologies in cancer research offer an unmatched opportunity to define cancer biology at many pathological and molecular levels [149], playing a key role in refining the diagnosis and recommending the treatment to the right patient.

Artificial intelligence based on big data, supported by machine learning, can be used to mine deep-level information in genomics, transcriptomics, proteomics, radiomics, digital pathological images, and other data, which can help clinicians to comprehensively understand tumors. The use of big data, AI, and machine learning in oncology ranges from detection and classification of cancer to molecular characterization of tumors and their microenvironments, to drug discovery and repurposing, and predicting treatment outcomes for patients [150,151].

Precision medicine has become an essential element of care for some cancers in various countries. Several national initiatives to deliver on the promise of precision medicine in oncology take place in the US, Australia, Israel, and higher-income countries in Europe [152,153], in which molecular profiling is available to comprehensively characterize a patient’s tumor, aiming to unveil its genomic makeup, which can inform the most effective treatment approach [152]. Biomarker-based precision medicine is now often considered the preferred standard of care for patients diagnosed with cancer in some countries, such as the US, given its value in selecting treatment for an individual patient and assessing the treatment response [147,148].

Further, with advancements in precision medicine, there will be increased reliance on dynamic biomarker testing across the continuum of cancer care, rather than testing tumor specimens just once, as they can more effectively track changes in cancer as the disease progresses. Managing patients effectively will require multiple types of biomarkers, often used in combination. Different biomarkers will be needed at various stages of the patient journey to ensure optimal care. This will influence existing guidelines and procedures in health services delivery, impacting health systems, payers, and reimbursement systems [148].

Biomarkers are essential to better classify patients by their probable disease risk, prognosis, and/or response to treatment [154], thus playing a crucial role in precision medicine. Biomarker-dependent drugs accounted for 42% of approvals by the US Food and Drug Administration (FDA) in the year 2018, compared to 28% in 2015, 27% in 2016, and 34% in 2017 [155]. In practice, only a minority of patients currently benefit from genomic testing to inform targeted drug treatment and targeted therapies, but this population continues to grow as the field advances [156].

Traditionally, cancer treatment would be tumor-type-centered, based on the location of the tumor in the body [157], but next-generation sequencing of advanced cancers has demonstrated that the genomic alterations vary and are not fully dependent on the tumor organ of origin, having complex and individually [158] unique genomic and immune landscapes [157].

The analysis of biomarkers plays a crucial role in the development of precision medicine [159]. Predictive biomarkers are used to determine if a patient will have a higher probability of a positive outcome using a specific drug, helping to guide treatment decisions and optimize therapeutic strategies [160,161]. Two categories of predictive biomarkers have been integrated into clinical practice: companion diagnostics and pharmacogenetic biomarkers [160].

Some genetic variations play significant roles in the development of various types of cancer [162]. The discovery of oncogenic driving genes led to the identification of biomarkers for cancer [163,164,165], which in turn facilitated the development of diagnostic tests and targeted therapies. Pharmacogenomic biomarkers can help distinguish those who will or will not respond to a drug, prevent adverse drug reactions, and support optimal drug dosing [164]. Up to 2024, nearly 600 pharmacogenomic biomarkers and nearly 400 drugs with pharmacogenomic biomarkers listed in the drug labels have been approved by the FDA [166].

Both predictive and pharmacogenomic biomarkers support the early identification of the pathology, in choosing the most appropriate treatment, and in monitoring the effectiveness of the treatment [167]. Some examples of predictive and pharmacogenomics biomarkers for cancer include ALK, BRAF V600E, BRCA1/2, EGFR, EZH2, GSTP1, HDAC, HER2, IGFBP3, and MGMT, to name a few [161,167].

Biomarkers have been widely used in clinical settings in developed countries. However, their broad application in LatAm is lagging, mostly due to costs, logistics, lab infrastructure, access to targeted therapies, and patient and biomedical education, as will be explained later in this study [168,169].

Advances in molecular profiling enable genomic classification of a patient’s tumor, leading to the development of targeted therapies, including tyrosine kinase inhibitors (TKIs), antibody–drug conjugates (ADCs), and immune checkpoint inhibitors (ICIs) [152]. As many oncological drugs have links to genetic variations and contribute to clinical outcomes, genetic testing is strongly recommended by medical societies in the US and Europe as part of the standard of cancer care within guidelines or general recommendations [170,171].

Precision medicine is advancing tailored diagnosis and treatments, optimizing efficacy and minimizing side effects. Some studies have illustrated the potential precision medicine has to improve health outcomes and relative cost-effectiveness, with a positive impact on health systems [172], and some small-scale observational studies have found evidence of improved health outcomes associated with precision medicine for cancer care [173,174,175,176]. The current application and potential of precision medicine is not equal across all cancer types [177,178]. Rather, it differs significantly due to the distinct biological complexities and genetic and non-genetic heterogeneity inherent to each cancer [179,180]. It is not feasible to address precision oncology in a generalized manner, and the development of precision medicine implementation plans must reflect this.

Precision medicine approaches have been used in clinical trials to analyze patients’ circulating DNA through liquid biopsy, as well as immune markers and other biologic features, to assess efficacy and make treatment decisions [157]. The recent and future advances in precision medicine in cancer include biotechnological advances and promising findings from recent clinical trials, with important research and development opportunities and gaps to be considered.

New strategies, some of which now have a proven track record, include gene-directed therapies and a host of immune-targeted approaches. The latter include checkpoint blockade, CAR T cell therapy, personalized vaccinomics, and the use of liquid biopsy [157].

- Checkpoint blockade: A type of drug that blocks proteins called checkpoints that are made by some types of immune system cells, such as T cells, and some cancer cells [181].
- CAR T-cells: Therapies made by collecting T cells from the patient and re-engineering them in the laboratory to produce proteins on their surface called *chimeric antigen receptors*, or CARs. The CARs recognize and bind to specific proteins, or antigens, on the surface of cancer cells to kill them. Currently available CAR T cell therapies are customized for each individual patient [182].
- Personalized vaccinomics: Vaccinomics combines the fields of immunogenetics, immunogenomics, immunoproteomics, and basic immunology to create vaccines that are tailor-made to an individual or groups of individuals. This broad range of omics applications to tumor immunology includes antigen discovery, diagnostic biomarkers, cancer vaccine development, predictors of immune response, and clinical response biomarkers [183].
- Liquid biopsy: Emerging technology that detects genomic information in bodily fluids and could alter traditional pathways of care for cancer. The technology is based on growing evidence that among certain cancers, tumor cells can release DNA into bodily fluids [184], known as circulating tumor DNA (ctDNA). ctDNA has been shown to harbor tumor-specific abnormalities, making it useful for diagnosis, treatment monitoring, and prognosis. It is increasingly used as a cancer biomarker, potentially contributing to improved clinical outcomes in certain cancer types, with ongoing clinical validation for different types of tumors, including non-small cell lung cancer and breast cancer [185].

A study from 2018 surveyed over 2000 specialists worldwide to understand which technologies were expected to be relevant to cancer care up to the year 2037 and found that liquid biopsy, genetic monitoring, and molecular imaging were the most cited ones [186].

A review of recent major advances in precision medicine for cancer care and the future implications of these advances, encompassing the end of 2022 through 2023, discussed novel approaches to drug design. It found that the advances have resulted in new precision oncology therapies that are proving successful in addressing several previously “undruggable targets” (target proteins whose functional interfaces lack defined pockets for ligand interaction) in the clinic, although quantitative figures of such increases are not presented [187].

#### 4.1.3. The Value of Precision Medicine

This section discusses the literature detailing the benefits of precision medicine in diagnosis and treatment, and how precision medicine helps cancer care to evolve, according to changes in clinical practice and real-world evidence.

Recent scientific breakthroughs and technological advancements spurred by precision medicine contribute to patient-centered care, as they improve the understanding regarding disease pathogenesis. This changes the way diseases are diagnosed and treated, with biomarkers and companion diagnostics enabling a shift from empirical medicine to patient-centered, targeted medicine [188].

Precision medicine plays a crucial role in oncology by customizing prevention strategies and tailoring treatments to individual patients based on their unique characteristics, such as genetic profiles. While the potential clinical benefits of precision medicine can be substantial, demonstrating these benefits in clinical practice can be challenging. Numerous studies have shown that matching patients to therapies based on the genetic makeup of their cancers can lead to improved patient outcomes, including increased progression-free survival (the duration from treatment commencement to cancer progression), higher response rates, extended time to treatment failure, and overall survival across various cancer types [189,190,191,192,193]. However, it is important to note that not all studies have shown consistently positive health outcomes for every cancer type [190,191]. Additionally, some statistically significant results may offer limited real-world applicability [189]. This emphasizes the necessity for ongoing investment in scientific research to fully harness the potential of precision medicine in oncology.

The evidence of the cost-effectiveness benefits of precision medicine is more elusive. Some studies report the cost-effectiveness of precision medicine for cancer [194,195]. Evidence indicates that precision medicine is at least cost-effective compared to usual care, but firm conclusions cannot be reached due to suboptimal study design and flaws in data handling [194,195]. In addition, patients receiving precision oncology therapies often require parallel treatments, such as chemotherapy, radiotherapy, toxicity management, and supportive care, which can significantly influence both the effectiveness and affordability of cancer care. Biomarkers that predict treatment response can help personalize decisions between modalities like chemotherapy and radiotherapy [196], while the toxicities associated with new targeted therapies must be actively managed [197]. Furthermore, supportive care plans are essential to minimize harm and optimize outcomes, addressing not only the tumor but the patient’s overall well-being [198]. The introduction of these parallel therapies should be considered in the discussion on the effectiveness and affordability of precision medicine.

Developing more robust cost-effectiveness studies is essential, with a focus on various types of cancer, as precision medicine impacts each tumor type differently. The success of implementing precision medicine can be demonstrated by evaluating the reduction in costs associated with ineffective treatments and the overall cost savings associated with precision medicine approaches [141]. Therefore, precision medicine holds enormous potential to revolutionize healthcare by significantly enhancing diagnostic accuracy, improving treatment efficacy, minimizing adverse effects, and ultimately proving to be cost-effective for individuals and healthcare systems alike. In addition, the use of biomarkers in precision medicine serves to guide treatment decisions based on individual genetic, molecular, or biochemical characteristics, thereby putting the patient at the center of care. There is an overlap of precision medicine and patient-centered care in practice [199], as the former leads to more clinical attention to the agency and conditions of patients. Precision medicine aims to guide targeted therapy and preventive procedures for those who would benefit from them, helping patients make informed decisions about their health and lifestyles, as well as involving patients in decision-making, including informed consent for genetic tests [199].

Biomarker testing in cancer involves profiling tumors or body fluids to detect changes in DNA, RNA, proteins, or other biomolecules [200]. There are many potential applications in cancer care, including risk assessment, screening, differential diagnosis, determination of prognosis, prediction of response to treatment, and monitoring of progression of disease, playing a critical role at all stages of the disease, and potentially even before the disease is clinically diagnosed [201].

Genomic biomarkers have been the most successful to date, but other biomarkers, including protein assays and transcriptomics, are being developed and tested [157]. An increasing number of genomic tests are available in practice, including tumor genome analysis for targeted treatment [130]. Specific clinically applicable tumor biomarkers (e.g., HER2, EGFR, and ALK) have been used to direct treatment for patients with targeted therapy [202]. Below are some examples of cancer biomarkers and targeted therapies approved by the US FDA.

- The human epidermal growth factor receptor (HER2) gene is overexpressed in 15% to 20% of breast cancers and in other cancer types such as gastric, colon, and head and neck. Drugs such as trastuzumab (ADC), pertuzumab (ADC), ado-trastuzumab emtansine (ADC), lapatinib (TKI), and trastuzumab deruxtecan (ADC) have been approved by the US FDA as targeted treatments.
- Epidermal growth factor receptor (EGFR) is expressed on the cell surface, with activating EGFR mutations commonly observed in patients with adenocarcinomas with no prior history of smoking, as well as in females and those of Asian descent, with targeted therapies including gefitinib, erlotinib, afatinib, dacomitinib, and osimertinib (all TKIs).
- Somatic rearrangements of the anaplastic lymphoma kinase (ALK) create common oncogenic fusions that lead to activation of different pathways in non-small cell lung cancer. Like HER2, ALK is a cell surface protein that regulates cell signaling pathways, and targeted therapy drugs include crizotinib, ceritinib, alectinib, and lorlatinib (all TKIs).

A more comprehensive list of commonly studied cancer biomarkers from different sample types is provided by the US National Cancer Institute [200,203].

Several studies have evaluated the approval process for new cancer medicines in LatAm countries in comparison to other regulatory agencies worldwide, such as the FDA and European Medicines Agency, revealing a lower number of drug approvals in LatAm and lengthy timelines for approval [204,205]. Studies also reveal limitations regarding access to targeted therapies in LatAm, even when these are approved by national regulatory agencies. Trastuzumab, for example, is approved in most countries in LatAm, but the drug is not generally available in public settings for the treatment of metastatic disease [206,207]. This also applies to the incorporation of biomarker testing approval.

#### 4.1.4. Policy Landscape of Precision Medicine and Oncology in LatAm

In this section, we provide an overview of the current regional and national policies and legislation related to cancer, whether precision medicine is integrated into them, and existing strategies and initiatives on precision medicine. This is important because regional and national action frameworks signal that an issue is on the political agenda and outline the intentions and strategies for addressing it.

Regional cancer plans act as strategic roadmaps for guiding interventions and policy formulation within countries. National cancer plans are important tools designed to reduce cancer incidence and mortality and improve the quality of life of cancer patients, making the best use of available resources [208]. Therefore, the policy landscape in a region and country plays a pivotal role in promoting advancements in healthcare, particularly through initiatives that support improvements in diagnosis and treatment outcomes, optimal resource allocation, and ultimately, improved patient care and public health outcomes, all of which could potentially be addressed by precision medicine. This section concludes with a brief overview of the precision medicine and oncology policy landscape in the US and Europe to provide a reference point.

At the regional level, PAHO lacks a comprehensive action plan or policy on cancer, but has developed the following:

- Plan of Action for Cervical Cancer Prevention and Control 2018–2030 [209];
- Knowledge summaries on breast cancer [210];
- Global Initiative for Childhood Cancer Working Group for Latin America and the Caribbean [211].

At both global and regional levels, neither the WHO nor PAHO currently offers a conceptual policy framework to guide the implementation of precision medicine, and the topic has received limited attention from these international organizations.

At the national level, Table 2 summarizes the current cancer policy landscape in focus countries. Notably, all five countries have up-to-date national cancer control plans, which include not only strategies for clinical cancer care, but also for research and development and surveillance efforts. The national plan for Colombia states its period of validity is 2012–2021; rather than developing another plan once that expired, the Ministry of Health and the National Cancer Institute decided to integrate the new Cancer Control Plan as part of the 10-Year Public Health Plan 2022–2031 [212]. Focus countries have also enacted legislation to support comprehensive cancer care by creating commissions for early detection of cancer and ensuring comprehensive treatment services, forming oncology units, and establishing/ensuring information reporting and systems. The continuous updating and implementation of national cancer care plans is necessary to address challenges and improve cancer care in LAC [32]. Beyond the five focus countries analyzed in this study, it is important to note that not all LatAm countries have national cancer control plans, nor non-communicable disease plans inclusive of cancer [213], and having a plan does not imply it is effectively implemented in practice. Furthermore, previous analyses have discussed that equity is not sufficiently integrated in national cancer control plans in the region [213].

Despite all national plans of the focus countries being issued after 2018, there was no mention of biomarkers or genetic markers in any of them, nor in supporting cancer legislation. Regarding precision medicine, the National Cancer Control Plan 2018–2022 of Argentina states that basic, clinical, and translational research can be improved to ensure timely diagnosis and adequate treatments, focusing on the general concept of precision medicine [214]. The National Policy for the Prevention and Control of Cancer 2023 of Brazil has an objective to incorporate more precise and less invasive diagnostic and therapeutic technologies [215]. The plans do not discuss precision medicine and biomarkers in detail and do not include provisions for their adoption and development. Overall, few countries in LAC have cancer control plans that include precision medicine because most were developed before there was enough evidence for the clinical application of precision medicine strategies [65]. No evidence was found to suggest that plans with a clear end date would include precision medicine in their successor plans. The existence of national policies and laws regarding precision medicine, or its inclusion in national cancer control strategies, can help advance the implementation of precision medicine in oncology at the domestic level [65]. Although not explicitly discussed in the literature, the lack of strategies in LatAm countries can hinder the development and adoption of precision medicine.

Although no specific legislation was found for precision medicine, some strategies and initiatives have been launched in LatAm with the goal of advancing precision medicine, acknowledging the potential benefits it might bring to patients, healthcare providers, health systems, and drug developers, and these are presented in Table 3.

A national precision medicine initiative was not identified in Panama or Colombia. In Argentina and Brazil, projects to map the genomes of the population have been implemented, with the objective of creating a genomic biobank, which is an organized form of storage for biomaterial resources and corresponding data, enhancing research capacity, training the healthcare workforce in precision medicine, and benefiting the local population [231,232]. The Argentinean government has also financed strategic projects through the Ministry of Science, Technology, and Innovation that aim to advance precision medicine, including the implementation of a second-generation massive sequencing platform to improve cancer treatments, and the development of an innovative R&D platform to facilitate the rapid translation of diagnostic products and services for oncologic clinical treatments [233,234]. The Ministry of Science, Technology, and Innovation of Argentina recognizes these projects as a natural consequence of recent advancements in genomic data generation and analysis technologies, combined with medical informatics, creating a personalized and efficient healthcare system [233].

In Mexico, the General Health Law includes provisions that ensure individuals own their genomes, prevent genetic discrimination, mandate explicit consent for genome studies, uphold confidentiality of genetic data, respect individuals’ preferences on genetic test awareness, and prioritize health protection in related research and innovation [235]. In 2023, a provision was submitted to the Mexican Government to include precision medicine under the General Health Law, promoting the use of precision medicine and genetic and molecular research [236]. The value of these initiatives is clear, reflecting the increasing global interest in precision medicine.

**Table 3:** Precision medicine initiatives in focus countries.

Country	Precision Medicine Policies, Programs, Research Projects, Laws	Issuing Authority	Main Objectives
Argentina	Reference Program and genomic biobank of the Argentine population (2021) [237,238]	Ministry of Science, Technology, and Innovation	Design, launch, and consolidate a genomic reference biobank and associated metadata of the Argentine population [231].
	National Biobank of Biological Samples (2017–2019) [239] Clinical genomics of pediatric diseases (2018–2019) [240] Argentine tumor genomics action map (2018–2019) [233] Development of a biotechnology platform for the application of Precision Medicine in Cancer and Uncommon Diseases in Argentina (2018–2019) [234]	Ministry of Science, Technology, and Innovation	Strategic projects financed by the Argentine government to establish a national biobank, develop and locally implement genomics technologies and protocols, implement a second-generation massive sequencing platform to improve cancer treatments, facilitate the rapid translation of diagnostic/predictive products and services for clinical treatments in the field of cancer.
Brazil	National Program for Genomics and Precision Health—Genomes Brazil (2020) [232]	Ministry of Health	Establish a reference genome for the Brazilian population, create a national database of genomic and clinical data, enhance scientific capacity and intellectual capital in genomic medicine, bolster the national industry for genomic products, and train the healthcare workforce in precision health.
Colombia	Law 2287—Regulates the operation of Biobanks and creates the National Biobank System (2023) [241]	Ministry of Health and Social Protection	Creates the National Biobank System and regulates the constitution, organization, and operation of biobanks in Colombia for the purposes of biomedical and technological research for the obtaining, use, processing, storage, transportation, and transfer of human biological samples, etc.
Mexico	General Health Law (LGS) (Last updated 2024) [235]	Ministry of Health	Provisions ensure individuals own their genomes, prevent genetic discrimination, mandate explicit consent for genome studies, uphold confidentiality of genetic data, respect individuals’ preferences on genetic test awareness, and prioritize health protection in related research and innovation.
Panama	No national precision medicine initiative was identified		

The precision medicine policy landscapes in the US and Europe are more advanced than in LatAm and consist of different programs, plans, strategies, and initiatives to foster the development and implementation of precision medicine. In the US, studies on colorectal and non-small cell lung cancer have found increasing and high biomarker testing rates among patients, although not all patients had equal access to it [242,243,244]. While guidelines exist, insurance coverage of biomarker testing varies widely [245], with existing socioeconomic inequalities in biomarker test utilization in the country [246]. In Europe, access to biomarker testing varies across countries, with higher access in countries with public reimbursement processes in place [247]. Northern and Western European countries have higher investment in healthcare, and this is reflected in higher access to biomarker testing, as well as quality, measured by participation in quality assurance schemes and with accreditation [247,248].

The Government of the United States has launched several precision medicine initiatives, including the aforementioned 2015 Precision Medicine Initiative [127,249]. In April 2018, the FDA issued two final guidelines to streamline next-generation sequencing-based tests [128]. The US National Cancer Institute has launched initiatives to support cutting-edge genomics research on adult and pediatric cancers to synthesize research in different fields of cancer genomics (structural, functional, and computational), improve patient outcomes, manage the enormous volumes of data generated by genomic characterization studies, compile information on clinical trials in precision medicine, and conduct research to facilitate discovery of molecular targets and translate those findings into the clinic [250,251]. The National Institutes of Health (NIH) aims to build a national research cohort of one million or more US participants [252] and also manages the Transdisciplinary Collaborative Centers for Health Disparities Research Program, which aims to explore the potential for precision medicine to promote health equity and advance the science of minority health and health disparities [253]. Other initiatives include consortiums, coalitions, and programs that promote the implementation of precision medicine [249].

Europe rapidly became a global leader in precision medicine due to centralized policies, extensive programs, and significant funding [254]. Key European Union-level policies focus on ensuring public health protection, covering standardization of medical practices, big data and information and communication technology, data sharing and cross-border interoperability, eHealth, innovation, healthcare sustainability, disease prevention, and patient engagement. Some European countries address precision medicine in their national regulations, plans, or strategies, aligned with the European Commission [254]. Italy advanced precision medicine through national plans on public health genomics and omics sciences, while the UK focused on genomics, personalized prevention, and citizen engagement. Estonia pioneered precision medicine implementation with innovation strategies and biobanking. Sweden, Denmark, and Finland emphasized genomics, biobanking, and data reuse, converging on eHealth and in the integration of genomics with data from registries and biobanks. Luxembourg and Spain have included precision medicine in their national strategies, including the importance of genomics and big data in health, data-driven healthcare, precision medicine training, and implementation of predictive medicine [254].

**Figure 1:**
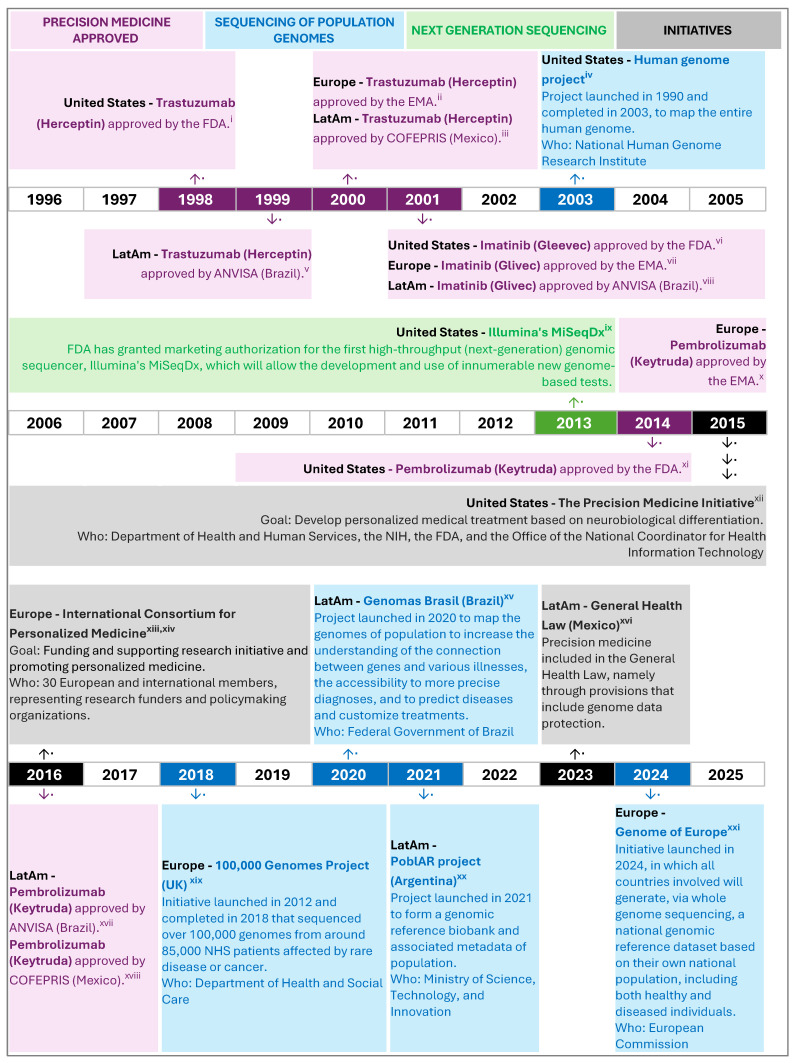
Timeline of key milestones of precision medicine in the United States, Europe, and LatAm. Sources: i. [258]; ii: [259]; iii: [260]; iv: [261]; v: [262]; vi: [263]; vii: [264]; viii: [265]; ix: [266]; x: [267]; xi: [268]; xii: [127]; xiii: [269]; xiv: [270]; xv: [271]; xvi: [235]; xvii: [272]; xviii: [260]; xix: [273]; xx: [231]; xxi: [274].

Figure 1 below presents a timeline of key milestones in the US, EU, and LatAm for precision medicine, genomic sequencing, and related initiatives. It shows the value and advantage of having a regional body oversee and drive the advancement and uptake of precision medicine and its related initiatives. Consider that even when a LatAm country (usually Brazil, sometimes Mexico, and in one instance, Argentina) experiences a milestone, it is only one of 33 countries in the region. Conversely, when the EU experienced a milestone, it propelled all 27 EU Member States (+/− other countries in the EEA and the UK—an EU member until 31 January 2020—that participate in, observe, or reference EU initiatives and regulations) simultaneously. Specifically, it shows that in 2015 and 2016, the US and the EU launched initiatives to fund and support research on and promote personalized medicine. No such region-wide or national-level initiative exists in LatAm to date.

Initiatives across Asia further demonstrate approaches to implementing precision medicine, which can serve as lessons for LatAm. In Thailand, a national initiative of genomics research (Genomics Thailand) emerged through a collaborative effort of governmental bodies such as the Health Systems Research Institute; Ministry of Higher Education, Science, Research, and Innovation; Ministry of Public Health; and Thailand Center for Excellence for Life Science, with the goal of establishing a comprehensive, population-level genome database and to facilitate the integration of genomics into national health policies [255].

In Singapore, the National Precision Medicine initiative is a whole-of-government, ten-year initiative, launched in 2017, that aims to establish precision medicine as a peak of research excellence for the nation, ultimately improving its health by identifying clinical applications that are cost-effective, sustainable, and relevant to Singapore’s communities. Key objectives include establishing a central coordinating entity, implementing population-scale genomics, developing robust governance frameworks for the responsible use of precision medicine, and creating innovative clinical pathways [256].

In Indonesia, although there is not a comprehensive legal framework on genomics yet, a decree of the Minister of Health of the Republic from 2022 concerns the implementation of the Biomedical Genome-Based Science Initiative for Precision Medicines and the Development of Genomics-Based Health Services for Certain Diseases, which carries out registry activities for patients with certain diseases, regulates biobanking, organizes the management of human whole genome sequencing examinations in Indonesia, and organizes the development of precision medicine [257].

In Malaysia, a national guideline on medical genetics service and/or genetic testing provides some degree of ethical oversight on the use of genetics in healthcare. In addition, medical societies promote awareness campaigns related to targeted oncology therapy for some types of cancer [275]. Finally, the regional research group Southeast Asian Pharmacogenomics Research Network has been established since 2012, including eight Asian countries, with the goal of enabling and strengthening pharmacogenomics research in the region [276].

In Africa, the H3Africa Initiative, funded by the National Institutes of Health (NIH) and the Wellcome Trust, supports innovative research into the genetic and environmental factors underlying diseases affecting African populations. Beyond funding research, H3Africa is enhancing research infrastructure by strengthening institutional capacities and establishing biorepositories to store biospecimens as shared resources. The consortium, which spans over 30 of Africa’s 55 countries and includes more than 500 members, has also developed robust policies for sample and data sharing to promote collaboration and maximize the impact of its findings, serving as an example of coordination and capacity-building across countries to advance genomics and genetics [277,278].

#### 4.1.5. How Precision Medicine Can Enable Equitable Cancer Care

This section presents literature relating to how precision medicine can promote health equity and the importance of equitable access to precision medicine. It highlights important factors to be considered to ensure that its potential impact can be harnessed.

It is important to note that precision medicine and health equity share a fundamental principle: providing patients with the specific care they need, rather than providing the same type of care for all, and both frameworks recognize that individuals’ health needs are shaped by unique biological, environmental, and social factors. Therefore, when made accessible to all, precision medicine has the potential to help deliver on the principles of health equity.

In practice, however, there is a lack of evidence detailing what countries with more advanced precision medicine landscapes have done to improve access. Most literature is theoretical, pointing to how precision medicine could increase health in/equity, rather than evidence-based studies and official data showing how different initiatives can narrow or widen the gaps in access and health outcomes. Table 4 presents three examples of initiatives that aim to ensure equitable access to precision medicine (but for which there was no evidence of outcomes), which could serve as useful guides for LatAm countries.

Despite the absence of definite outcomes reported in the literature, precision medicine has demonstrated the potential to improve health outcomes with relative cost-effectiveness [101], meaning that the potential for improved care due to the adoption of personalized medicine could prove beneficial and outweigh the challenges it faces [281]. Many actions required to prescribe precision medicine treatments occur in reference labs, involving the logistics of moving only samples, rather than patients. The implementation of PM does not require laboratory infrastructure in each city, but it does necessitate the development of a service infrastructure with access to experts through partnerships, the ability to transport samples and conduct tests, and the presence of centers of excellence. Thus, the logistical and financial barriers to delivering care could be lower, making it easier to reach those who are most disadvantaged, as opposed to using other therapies that require in-person testing, more expensive equipment, and complicated processes. Furthermore, the challenges remain the same as those for other therapies, but with the added benefit of greater response rates for targeted therapies.

The impact of inequitable reach and quality of cancer care manifests as disparate cancer outcomes in LatAm. People on low incomes, with low levels of education, from ethnically diverse backgrounds, and/or living in rural areas experience disproportionate disease burden and lower survival [282]. In addition, lower education levels are associated with lower health literacy, which negatively impacts the acceptability of precision medicine approaches and consequently, their uptake [283]. Precision medicine has the potential to improve care quality and cancer outcomes better than traditional approaches because of its targeted nature. That is, precision medicine is concerned with the biology of a person rather than a person’s socioeconomic status.

The use of genomic data and AI can support oncologists’ prognostication and therapy response predictions through optimizing, harmonizing, and adopting digital solutions in electronic health records, and can potentially promote equity by reducing differences in quality of healthcare decision-making, improving case management, and increasing uptake of novel treatments [59,284]. Artificial intelligence can also be integrated with telemedicine to enhance healthcare access in remote areas [285]. For AI to be effective, high-quality data, data sharing, and a robust ethical regulatory framework are essential. These measures help prevent health inequities by ensuring that AI systems do not perpetuate biases present in the data [286]. In LatAm, AI is still in the early stages of implementation, with some studies being conducted with the aim of improving its performance in the region [59,110]. The future of precision medicine will rely heavily on AI and machine learning, which are crucial for analyzing the vast amounts of genomic data currently beyond our capabilities. Precision medicine also presents opportunities for addressing health equity issues without stigmatizing individual patients, as it uses aggregated biological and genetic non-identifiable data to advance medicine in the diagnosis and treatment of diseases [281], eliminating any potential for bias based on race, religion, or socioeconomic status.

Despite its potential, the ways in which precision medicine will either address or exacerbate equity challenges overall are still not clear [282]. What is clear is that a policy framework is needed to guide intentionally equitable uptake of precision medicine, or we risk a future where precision medicine not only fails to address existing health inequities but could potentially exacerbate them.

Access to prevention, diagnosis, treatment, and care includes a combination of psychosocial and structural factors, such as availability, accessibility, and acceptability. Some groups experience lower access to and utilization of health services and specialty care, including genetic services, leading to lower rates of diagnosis, suboptimal care, and worse health outcomes [10]. Healthcare systems in LatAm are characterized by a lack of coverage for populations excluded from social security or other public financing mechanisms [287]. There is unequal access to precision medicine between the private and public health systems, with more precision medicine taking place in the private sector [169], further accentuating healthcare inequities due to socioeconomic levels [101].

Healthcare systems require adequate infrastructure, resources, optimal regulations, and scientists with experience in precision medicine technologies to implement it in practice [110]. In addition, systems must be equipped with multidisciplinary teams comprised of clinicians, pathologists, molecular specialists, and others, all working together in a highly coordinated clinical setting for the adequate implementation of precision medicine and companion diagnostic testing. In Brazil, for example, such integrated teams are usually unavailable due to the short supply of both financial and human resources [288]. Strategies must be implemented to bring awareness and education to public and private payers on the potential benefits of precision medicine, to help build the case for improved availability and an efficient drug approval process [288].

Innovative technologies can promote health equity given their enormous potential to improve human well-being [289]. For that to be realized, innovation must have equity at its core, where the goal is to improve outcomes for all groups [290]. However, the introduction of innovations is subject to barriers regarding uncertainties around production costs, development, availability, and economic difficulties for their incorporation into health systems. In Brazil, for example, these issues are exacerbated by supply, distribution, and access inequalities for cancer diagnosis and treatment services and technologies [291].

Conditions of reimbursement of health systems play an important role in access to precision medicine tools, such as molecular testing. In Brazil and Colombia, for example, private and public healthcare systems only reimburse germline genetic testing ordered by certified geneticists, genetic counselors individually, or as a part of a multidisciplinary tumor board, which imposes an additional challenge given the shortage of these specialists, as reported in a study from 2019 [101], and can promulgate inequities. In Brazil, a review from 2023 indicates that the presence of specialized personnel can be an advantage for the implementation of NGS technologies in the country, but there is a lack of specialized personnel to deal with the complexity of the data management required for precision medicine. In fact, there are significant limitations on the interpretation of NGS tests by community oncologists or general physicians, and this is also a challenge in more developed countries [292]. As previously stated, in Brazil and Argentina, where the state must provide comprehensive access to healthcare by law, patients may use litigation to access drugs, which can also increase inequity in treatment access, as lower-income citizens cannot afford the legal costs [109]. The literature on Panama’s experience with the judicialization of the right to health is scarce [293], but there is evidence of cases where the duty of the State in medical and health care was brought to court [294].

The introduction of innovative technologies and approaches, such as precision medicine in cancer, depends on the health systems and regulatory aspects of the policy landscape. In the case of lung cancer in LatAm, access is hindered by a very bureaucratic drug regulatory approval process, expensive targeted therapies, and a lack of widespread access to genetic diagnostic tools [295,296].

Editorial and comment pieces published in the Nature journal in 2021 have discussed the need for a health equity agenda in genomics research [9,297]. The discussion regarding health equity encompasses the need to address the underrepresentation of minority and ethnic populations in genomic research, as diverse racial and ethnic backgrounds are needed in precision medicine studies to produce robust evidence [87]. Further, the lack of representation in the data used to build precision medicine models is likely to limit their impact in the real world, particularly for diverse populations, such as those in LatAm [9]. Cancer clinical research must be oriented to establish cancer biomarkers adapted to specific populations according to their ethnicity, allowing the improvement of patient outcomes. However, to ensure that precision medicine can lead to improved health outcomes for all segments of the population, a health equity agenda needs to go beyond basic and clinical research [10].

Patient organizations and healthcare professionals acknowledge that precision medicine should take into consideration that access equity, and evidence of diagnostic accuracy and improved clinical outcomes, come with risks of overpromising and underperforming [129]. The implementation and adoption of precision medicine requires the alignment of appropriate education, data systems, coverage and reimbursement policies, health system processes, and health policies [298].

International scientific evidence of clinical validity and utility of precision medicine is needed to inform the necessary local calculations of cost-effectiveness to guide reimbursement procedures [299]. Robust cost-effectiveness studies are lacking in LatAm, and the fragmentation of healthcare systems adds significant complexity to conducting this type of research. However, analyzing the cost-effectiveness of precision medicine at both regional and national levels is crucial to identify which interventions are feasible according to resources and health systems context [59]. It must be highlighted that, while most references are peer-reviewed, evidence gaps remain, either due to limited data from certain countries or regions or due to publication bias. In the field of precision medicine and biomarker research, this bias can distort the scientific literature, overstate progress, and ultimately influence research priorities and clinical decision-making, which may affect patient care [300]. The implementation of precision medicine will depend on its ability to demonstrate and convince decision-makers of its value, and whether payers and consumers are willing to pay for targeted therapies. There are still challenges in assessing the value of precision medicine relative to its costs and whether payers should reimburse for testing [298]. Which payers (private and/or public) decide to pay for precision medicine will have a huge impact on how equitably its associated benefits are distributed in society.

### 4.2. Literature Review of Policy Gaps and Challenges Implementing Precision Medicine in LatAm

Evidence indicates that precision medicine is a powerful approach to improving cancer prevention, diagnosis, and treatment, with further potential benefits being possible as the field advances. However, there are challenges and barriers to the adoption of precision medicine for cancer in LatAm that must be considered when envisaging the potential impact it can have on cancer care. Based on the literature, both from a policy and scientific perspective, challenges and barriers can be categorized into those related to (1) scientific development of precision medicine, (2) capacity to implement precision medicine in healthcare delivery services, (3) economic barriers, and (4) policies and regulations for implementation of precision medicine. Below, we present key policy gaps and challenges related to the uptake and equitable application of precision medicine in LatAm from the perspective of these four categories.

#### 4.2.1. Challenges Regarding Scientific Development of Precision Medicine

The overall cancer profiles in LatAm countries vary significantly due to genetic mixtures between ethnic groups and different lifestyles among populations [13], given that somatic and germline mutations have a great impact on cancer prognosis and/or response to therapy [13]. The genetic heterogeneity of LatAm’s population can complicate the implementation of precision medicine in the region [169], given that precision medicine relies heavily on data, analytics, and genetic information [298]. On the other hand, it may represent a good argument for implementing precision medicine in the region, as the heterogeneity of the population could drive its adoption.

The genetic sequencing of patients in different countries and regions is necessary to properly characterize variations and biomarkers, and consequently, the development of new pharmacogenomic therapies specific to certain populations [13,301]. An analysis from 2016 indicated that less than 4% of samples analyzed in genome-wide association studies conducted up to that year were from individuals of African and Latin American ancestry, Hispanic people, and Native or Indigenous peoples [302]. In addition, there is scarce representation of Latin American patients in biobanks [303]. The lack of representation among biomedical research participants limits both the generalizability and availability of genomic-based treatments or prevention strategies [304]. Diagnostic/therapeutic strategies should be adapted to each population and take into consideration the relationship between ethnicity and types of biomarkers in each population [13,169], but the lack of genomic representation of LatAm populations in studies [169] represents a challenge for precision medicine efforts. Furthermore, the underrepresentation of LatAm communities and lack of representation in international databases could potentially serve as a justification for decision-makers to refrain from implementing precision medicine in the region.

Clinical trials are essential to understand the effectiveness and safety of targeted approaches, ultimately advancing the field of precision medicine. However, the number of oncology clinical trials related to targeted therapies and molecular profiling in cancer in LatAm is drastically lower than in Europe and the US [65,305]. Most oncology trials in the region have been sponsored by pharmaceutical companies, and clinical investigation commonly grants access to both standard and experimental drugs [109], which highlights the need for increased funding from governments and relevant foundations [305]. On a positive note, LatAm does have some modern medical facilities, well-trained investigators, and premier research groups, with large academic centers in international research collaborations [109], which can contribute to capacity-building for high-quality research participation in the region.

Recent clinical trials with targeted therapies have shown promising response rates. However, as patient selection becomes increasingly precise, biomarker-positive populations can be very small, limiting the potential number of patients that would benefit from the most effective targeted therapies for specific tumor types, leaving most patients without access to such treatments [178]. Patient selection and biomarker testing standards must adapt, together with clinical trial designs, as the complete genomic landscape of a tumor or the evolutionary plasticity of solid tumors are not often considered [306]. Future research and development should prioritize enhancing the sensitivity and specificity of existing cancer diagnostic methods, identifying cost-effective alternatives, integrating multi-omics data preclinical experiments and clinical trials, and validating emerging techniques to establish their efficacy in cancer detection [178,307].

It must be acknowledged that despite the growing number of recent clinical trials of targeted therapies that have shown promising response rates, it is still hard to demonstrate the clinical benefit of precision medicine across all cancer types, with findings of studies indicating minimal gains, or with limited practical implications to factors such as small effect sizes, variability in study design, or differences in patient populations [189,190,191].

Biomarkers’ adoption in clinical practice faces challenges, meaning they are not widely implemented, nor is their development the same across cancer types (e.g., the development, regulatory approval, and clinical implementation of biomarkers are significantly more advanced for breast cancer than for pancreatic cancer) [308,309]. The development and validation of biomarkers is scientifically and economically challenging, and it is difficult to implement any biomarker in clinical practice, which is partially due to tumor heterogeneity and inter-laboratory differences in pre-clinical findings [147]. It is particularly challenging to identify those biomarkers that reliably predict specific disease-related clinical outcomes based on clinical applicability, given that diseases are heterogeneous and complex, and it may not be possible to find one surrogate endpoint for each disease [310]. Biomarker research focuses on two aspects: clinical validity and clinical utility. Clinical validity refers to robust evidence demonstrating that a tumor biomarker test can stratify a population into distinct groups based on biological or clinical outcomes, with statistical significance [311]. For example, in genetic testing for multiple endocrine neoplasia type 2 (MEN2), clinical validity refers to robust evidence demonstrating that testing for RET mutations can accurately stratify a population into distinct groups based on their risk, with statistical significance, enabling early detection of affected family members before symptoms develop [312]. In contrast, clinical utility is established when high-quality evidence shows that using the tumor biomarker test improves clinical outcomes or achieves equivalent outcomes at a lower cost or with reduced toxicity [311]. For example, in genetic testing for multiple endocrine neoplasia type 2 (MEN2), it refers to the risks and benefits resulting from genetic test use, in which RET mutation testing provides a means to identify individuals who would benefit from preventive surgery [312].

However, prospective trials to gather clinical utility data are time-consuming and costly. Consequently, there are fewer approvals based on trials where determining the clinical utility of a specific tumor biomarker test is the primary objective [311].

Biomarker testing reports must be interpreted by an expert or a team of experts (including medical oncologists, surgical oncologists, pathologists, basic scientists, pharmacists, clinical nurses, physician assistants, and genetic counsellors) to select the optimal treatment to be prescribed and administered to the patient. In addition, treatment decisions consider coverage and reimbursement aspects [148,313,314,315].

The use of biomarkers to guide treatment decisions faces challenges, such as insufficient tumor tissue for analysis, prohibitively long turnaround times, and inconsistent availability of results [314]. Nonetheless, biomarkers have been increasingly included in different therapeutic areas within FDA- and EMA-approved drug labeling [316,317], indicating that their incorporation in clinical practice is increasing despite these barriers and challenges.

#### 4.2.2. Challenges Implementing Precision Medicine in Healthcare Delivery Services

Overall, the provision of cancer care in health systems in LatAm faces major limitations regarding access to trained healthcare professionals, access to new therapies, and adequate facilities for cancer care, due to inadequately distributed budgets across locations and geographic and cultural barriers [110].

One of the main challenges associated with the capacity to implement precision medicine in healthcare delivery services is the limited availability of adequate infrastructure and trained human resources to perform genetic testing and molecular profiling and to interpret results. There is a limited number of certified laboratories that perform such tests [65] and a lack of specialist oncologists trained to perform and interpret advanced molecular tests due to a lack of trained personnel and scarce bioinformatics support [168]. Efforts are needed to harmonize laboratory standards and establish comprehensive training programs for preparing the next generation of laboratory professionals [65].

The interpretation of biomarker testing reports poses significant challenges [314], highlighting a critical need for improved physician education in this area. Understanding the specifics of the available tests, selecting the most appropriate test for a given clinical scenario, and accurately interpreting the results are essential competencies for healthcare providers. These skills are often lacking and represent key unmet needs.

The adequate implementation of precision medicine and companion diagnostic testing requires a multidisciplinary team comprised of clinicians, pathologists, molecular specialists, and others, but such teams are usually unavailable due to the short supply of both financial and human resources [288]. For example, genetic testing is available in Brazil, but its use is greatly restricted, as only some laboratories have the human or technical resources to perform high-quality testing [288]. In 2010, the CDC defined genomic competencies required from healthcare workers, and one of them is to “Identify the role of cultural, social, behavioural, environmental and genetic factors in development of disease, disease prevention, and health promoting behaviours; and their impact on medical service organization and delivery of services to maximize wellness and prevent disease” [318].

Precision medicine requires a stable logistical infrastructure for the cost-effective and efficient transport of biological samples to molecular laboratories, but these are usually lacking [168]. Tests are often sent abroad for analysis [168], with data being kept on the laboratories conducting the testing. Furthermore, laboratory infrastructure and specialized oncologists are mostly concentrated in urban centers [169,319]. These issues hamper the equitable implementation of precision medicine for cancer in the region [319]. The implementation of precision medicine is also hampered by insufficient funding for the development of computing, AI, and machine learning to support clinical judgment and managing processes, both of which require improvements in health workforce capacity-building [59].

Finally, significant challenges remain regarding data reliability and the notable shortage of information on cancer care and control in the region. Many countries lack population-based cancer registries and adequate histopathological reporting systems, limiting access to accurate epidemiological data, which hinders efforts to understand the true cancer burden, plan effective control measures, and develop strategic policies for resource allocation. The registries are crucial for understanding the cancer burden and planning effective control measures, as they provide essential data for strategic cancer management [32]. Reliable data, including tumor information and cancer prevalence, is important to justify robust health policies and guide decision-making processes, thus impacting the implementation of precision medicine. In fact, the development of precision medicine could be an incentive for such countries to develop/strengthen their registries. LatAm also faces a significant lack of data on the use of genetic tests, such as who is using them, where, how, and for what purpose. Similarly, there is little information about precision medicine projects and studies in the region, including details on who is conducting them, their focus areas, and funding sources.

#### 4.2.3. Challenges Regarding Economic Issues

There is an issue of equity in access to molecular diagnosis and treatment in LatAm, due to costs [319]. Most low-income populations have limited access to cutting-edge genomic technologies and to next-generation anti-cancer targeted drugs, mainly due to unaffordability. Healthcare systems in LatAm are characterized by a lack of coverage for populations excluded from social security or other public financing mechanisms [287], with fragmented and under-financed public health systems leading to healthcare inequities due to costs [319].

Under-financing of public health systems leads to inequalities in access to precision medicine between the private and public health systems, with incorporation of precision medicine taking place in the private insurance sector [169], further accentuating healthcare inequities due to socioeconomic levels [101]. For instance, the two-tiered healthcare delivery system in Brazil hampers effective and equitable implementation of precision medicine in cancer, as there is a discrepancy in tests and treatment available between people covered by the private and public healthcare sectors [288].

Conditions of reimbursement play an important role in access to precision medicine, and therefore, in the equitable uptake of it [65]. Therapeutic options are not often reimbursed, leaving patients to face very high out-of-pocket costs [101]. Regarding molecular testing, private and public healthcare systems in Brazil and Colombia only reimburse germline genetic testing ordered by certified geneticists, genetic counselors individually, or as a part of a multidisciplinary tumor board, which poses an additional challenge given the shortage of these specialists [101]. Regarding tumor testing, the absence of national protocols for tumor tissue handling that adapt the ASCO/CAP recommendations to the national realities in LatAm challenges the standardization of collection, handling, transportation, and processing of specimens [101]. Further economic challenges that limit the implementation of precision medicine in the region relate to the costs of required infrastructure and drugs, despite the potential cost savings related to positive health outcomes [101]. Oncologists hesitate to prescribe targeted therapies because health insurance does not always cover them [65]. This is a common challenge, with oncologists questioning the value of testing when the treatment is either unavailable or unaffordable for the patient. Furthermore, costs of precision oncology drugs are usually perceived as higher than non-targeted drugs [65]. These arguments can be countered by emphasizing that selecting the right treatment for the right patient reduces the number of individuals receiving unnecessary therapies, minimizes toxicities, and improves outcomes. However, to strengthen this point, it must be supported by robust evidence and clear examples.

High-cost drugs are often inaccessible in LatAm countries, and patients often face out-of-pocket costs [101]. Cancer drugs usually have a higher price in many low- and middle-income countries when compared with higher-income nations [320], or, at times, the prices are lower but still too high when adjusted for purchasing power and average incomes, and there is often no relationship between drug prices in low- and middle-income countries and their gross domestic product [65]. A study comparing cancer medicine affordability differences between countries found that less than five days of wages were required to purchase one defined daily dose of cancer medicines in upper-income European countries, while in LatAm, it required a median of 10.53 days to purchase one defined daily dose [320]. Anti-cancer drugs are often deemed not cost-effective in LatAm, as illustrated by a study that estimated that for trastuzumab to become cost-effective, it would need to have its price dropped between 69.6% and 94.9%, according to the WHO threshold [321]. Finally, not all indicated targeted therapies are commercially available in LatAm, and not all patients can access therapies currently approved [101]. These factors pose significant challenges to conducting cost-effectiveness analyses in LatAm that would yield positive results.

Public investment in cancer research remains limited in LAC, where the average share of gross domestic product allocated to research and development is just 0.7%, compared to 1.7% globally, 2.1% in East Asia and the Pacific, and 2.5% in North America and Europe [322]. Increased investment in clinical research is essential, as it serves as a key pillar for innovation and progress that can raise awareness and promote education of both patients and the medical community. Prioritizing this field in the region requires streamlined regulatory processes and enhanced international collaboration, particularly to advance the discovery of biomarkers relevant to LatAm populations [59].

The underfunding of public health systems and insufficient investment in precision medicine can stem from the limited capacity of resource-constrained countries to generate economic resources. This creates challenges in setting priorities, which often results in political challenges, particularly a lack of political will. This hesitation by decision-makers is frequently based on the perception that precision medicine is too expensive [65], without considering its potential to promote health equity. LatAm countries face shared challenges such as political instability and corruption [65,323], which can result in severe financial restrictions that destabilize health systems and disrupt cancer care quality [32]. Political and economic issues are often interconnected in the region, leading to suboptimal resource allocation and underfunding, but there must be strong political will to address and prioritize the growing burden of cancer [323], including by investing in efficient approaches, which include precision medicine.

#### 4.2.4. Challenges Regarding Policies and Regulations for Implementation of Precision Medicine

Currently, there is an absence of provisions that can support the comprehensive implementation of precision medicine for cancer in the focus countries. There are limitations to accessing targeted therapies, partially derived from delayed approval and registration processes for both broad access and clinical trials [288,319]. Overall, new technologies take longer to be incorporated in LatAm than in high-income countries [65]. The time to regulatory approval of innovative drugs in the region varies from 730 days from FDA approval in Brazil to 1184 days in Colombia. Even once approved, these medicines take additional time to be available in public healthcare systems, varying from 156 days from local approval in Argentina to 1215 days in Mexico [324].

The introduction of innovations is subject to barriers regarding uncertainties in production costs, development, availability, and economic difficulties for their incorporation into health systems. In Brazil, these issues are exacerbated by inequalities in supply, distribution, and access to services and technological resources for cancer diagnosis and treatment [291]. It must be recognized that in the context of limited resources, such as in LatAm, guaranteeing access to standard therapies may be a higher priority than access to new drugs.

Compliance with law, regulatory pathways, and safety planning can be challenging for precision therapies and diagnostics [6]. Regulatory decisions should be grounded in assessments of quality, efficacy, and safety [325]. In LatAm, significant challenges remain regarding the development and implementation of health technology assessment policies [326], and regulatory decisions in the region are often closely tied to coverage and reimbursement decisions [325]. Ideally, these processes should be independent to ensure that scientific evidence guides regulatory decisions and the adoption of health technologies.

Targeted therapies may be subject to regulatory lags. Out of 228 new products approved globally between 2014–2021, only 130 (57%) were approved in LatAm, and within countries, the number varied: 22 in Peru, 68 in Argentina, 65 in Colombia, 63 in Brazil, and 56 in Mexico. Although Argentina had the most medicines approved, it only had 1 fully available in the public system, while Colombia had 35, and Mexico, 24 [324]. Not all of these products are precision medicines, but it is likely that a significant portion of these innovative products fit that description.

Global collaboration is necessary to establish standardized experimental and reporting methods, as well as clinically relevant cutoffs in biomarker analyses, allowing findings of different studies to be used to produce robust evidence, ensuring that as many patients as possible can benefit from precision-based treatments [178]. Communication between the industry, academic and scientific community, governing bodies, payers, as well as patients and families is needed to ensure better collaboration among stakeholders, more visibility of precision medicine, shared and informed multi-perspective decision-making, effective policy development, enhanced education and knowledge on the topic, and potentially increased funding; thus, it is imperative to improve awareness and literacy regarding precision medicine [178].

## 5. Discussion

Cancer represents a significant public health issue with substantial societal and economic costs, which is especially concerning given its rising incidence and high preventability, highlighting the urgent need for comprehensive strategies for prevention, early detection, and effective treatment, both globally and in LatAm.

Cancer care in LatAm faces significant equity challenges. Fragmented healthcare systems, unequal healthcare coverage, budget limitations, healthcare workforce shortages, geographical and cultural barriers, and inadequate access to new therapies and technologies impact patient outcomes. In addition, equities affect all stages of cancer care, from risk factor exposure and prevention to early detection, treatment, and palliative care, especially given the differences in access to and quality of care between the public and private sectors. Achieving health equity in cancer—where everyone has an equal opportunity to prevent, detect, and receive proper cancer care—currently seems a pipedream.

Precision medicine holds enormous potential to improve preventive care, enhance diagnostic accuracy, deliver more effective treatments, improve patient-centricity, reduce side effects for individuals unlikely to benefit from certain therapies, and possibly be a cost-effective approach for cancer care. Therefore, if precision medicine is implemented in health systems in an intentionally equitable way, the use of biomarkers can help achieve health equity by providing care tailored to genetic, molecular, and environmental profiles, ensuring all patients receive the most appropriate and effective care, regardless of their socioeconomic or other status. Equitable and patient-centered implementation of precision medicine can empower patients, fostering trust and engagement between patients and healthcare providers.

Evidence regarding the adoption of precision medicine for cancer in LatAm remains limited, yet it indicates that there are inequities in access to biomarker testing. These inequities directly impact the potential benefits that precision medicine could offer, highlighting the urgent need for improved access and equity in healthcare delivery across the region. Precision medicine initiatives are currently driven and funded primarily by the pharmaceutical industry [59,65], with some established biomarkers becoming widely used due to early efforts by pharmaceutical companies, which often fund campaigns and provide diagnostic tests at no cost to encourage their adoption. However, it is essential for countries to begin developing their own capabilities. Future research and developments in the region should prioritize identifying cost-effective applications for precision medicine, integrating multi-omics, and validating emerging techniques to establish their efficacy in cancer detection in LatAm countries.

Precision medicine is a rapidly growing movement with the potential to transform all areas of medicine. However, its adoption must be guided by structured policies to ensure an organized and equitable integration into healthcare systems. Without such frameworks, precision medicine risks penetrating different areas of healthcare in a disorganized manner, likely exacerbating existing inequities. Although markets may not yet be fully prepared for this fast-evolving approach, much like with other medical innovations, strategic and systematic implementation is crucial. It is likely that in the early implementation stages, precision medicine would widen inequity, as it might be limited to those who can pay for it or are close to it, but in the long term, it would improve equity, as patients would have more access to better treatment. Therefore, it is crucial to implement precision medicine in a strategic and systematic way to ensure that its potential to improve health equity is addressed.

Access to testing alone will not immediately guarantee that patients receive treatment, but it is essential to prioritize equitable availability of testing for patients across all regions of the country. Additionally, robust monitoring and evaluation mechanisms are vital to measure the impact of precision medicine on health systems. Equally important is ensuring the economic sustainability of policies, balancing innovation with affordability and inclusivity. Whilst beyond the scope of this research, it is also vital to consider the diverse perspectives of the populations impacted most by the lack of access to cancer therapies and precision medicine. This presents an important opportunity for further exploration.

The challenges to the implementation of precision medicine for cancer care in the region require immediate and significant investments in order to achieve the following:

(a) Explore the impact and implications of genetic heterogeneity in LatAm populations and address the low number of oncology-related clinical trials related to targeted therapies in the region.
(b) Build adequate infrastructure and facilities for cancer care, train health professionals, and better distribute resources and/or deliver virtual consultations, when possible, to address geographic and cultural barriers whilst optimizing patient-centricity.
(c) Sustainably finance public health systems, simplify reimbursement processes, and address issues of affordability and availability of targeted anti-cancer drugs in the public health system.
(d) Ensure that regulations and policies keep pace with science to support the comprehensive implementation of precision medicine for cancer, including compliance with laws, regulatory pathways, and patient safety.

The current policy landscape in the LatAm focus countries is not conducive to addressing the existing challenges across the continuum of care and health systems, and the benefits of precision medicine and biomarker testing will not be enjoyed equally unless intentional policies are developed now to ensure that the benefits can be widely distributed to all who need them.

In the focus countries, it is important to include precision medicine in the next iteration of national cancer plans and strategies, as governmental policies supported by scientific evidence can guide cancer control strategies nationally, and countries that have higher implementation of precision medicine for cancer have local policies/laws [65]. The complex, time-consuming, and bureaucratic approval and registration processes for both market authorization and clinical trials should be the subject of reform in the region.

Policy improvements depend on collaborative efforts among stakeholders such as scientific societies, governments, payers, healthcare providers, patient groups, research centers, and pharmaceutical companies. These collaborations are crucial to help drive precision medicine’s evolution beyond tissue or receptor types, toward truly patient-centered care based on the patient’s preferences, characteristics, and their complete health state, and for conducting research, generating evidence, developing and commercializing technologies, and establishing strategic directions and regulatory frameworks to ensure equitable access to innovations and maximize the benefits of precision medicine in cancer care for those who need it.

Overall, the provision of cancer care in health systems in LatAm faces major limitations regarding access to trained healthcare professionals, access to new therapies, and adequate facilities for cancer care, due to inadequately distributed budgets across locations and geographic and cultural barriers. Until these structural and resource-related issues are addressed, it is unlikely that cancer care can be equitably distributed, but hope remains that—with new and intentionally equitable, forward-thinking policies—precision medicine might herald a new opportunity to improve equity in cancer care in LatAm.

Since healthcare systems in LatAm are characterized by a lack of coverage for populations excluded from social security or other public financing mechanisms, the question of “Which payers (private and/or public) decide to pay for precision medicine?” will have a huge impact on how equitably its associated benefits are distributed.

Given that the WHO’s recommended actions to improve health equity (see Section 3.3) call on evidence-informed action (among other things) to ensure high-quality and effective health services are available, accessible, and acceptable to everyone, everywhere, when they need them [8], it is valuable to consider what this would look like for precision medicine in oncology. The WHO has also promoted equity and global access to genomic data, outlining globally applicable principles designed to guide stakeholders in the responsible collection, use, and sharing of human genome data [327].

## 6. Recommendations (Suggested Solutions)

Table 5 summarizes key challenges and gaps related to cancer, precision oncology, and equity in LatAm, as identified during our literature review, along with suggested solutions to address them. Note that these suggested solutions are intended to offer a starting point for discussions on how stakeholders can work together to address the gaps and challenges inhibiting equitable access to precision medicine in LatAm and stymying precision medicine’s potential to improve health equity in cancer care in the region. The effectiveness and feasibility of these suggested solutions are likely to vary across countries and depend on context-specific factors, such as societal structures, genetic diversity, and varying degrees of social equity; thus, their implementation should be adapted to local contexts and supported by further empirical evaluation.

## 7. Conclusions

Multiple complex problems that persist within cancer care, its reach, quality, and outcomes are reported in the literature, alongside evidence that access to precision medicine offers many real and potential benefits to diagnostic, treatment, and care quality and outcomes. The current policy landscape in LatAm is not conducive to improving the reach, quality, or outcome-related problems in cancer care, nor to realizing the full potential of precision medicine. Further, the precision medicine landscape and broader health system and policy landscape in LatAm are lagging significantly behind other regions, emphasizing the fact that precision medicine policies are not where they should be and that there is a need for policy frameworks that extend beyond regulation and into access-related components for precision medicine.

Precision medicine has the potential to reduce health inequities in LatAm by improving health outcomes for all people living with cancer through accurate diagnosis and targeted treatment. However, for it to achieve this goal, it must first be implemented in health systems and applied in a way that is intentionally equitable.

The time is now to develop an intentionally equitable policy framework to guide broad and deep applications of precision medicine in LatAm countries and to improve health outcomes for all who need it, not just the lucky few who can afford to pay for it.

## Figures and Tables

**Table 1:** Impact of cancer in focus countries: Argentina, Brazil, Colombia, Mexico, and Panama.

Country/ Region	Cancer Incidence New Cases (2022)	Cancer Incidence Age-Standardized Rate Per 100,000 (2022)	Cancer Prevalence 5-Year Prevalence (2022)	Cancer Mortality Number of Deaths (2022)	Cancer Mortality Age-Standardized Rate Per 100,000 (2022)	Disability-Adjusted Life Years and % of Total DALYs (2019)
Global	19,976,499	212.6	53,504,187	9,743,832	109.8	251,390,451.29 9.93%
Latin America and the Caribbean	1,551,060	199.9	4,096,032	749,242	96.5	16,470,687.03 9.94%
Argentina	133,420	231.8	395,958	70,251	123.6	1,977,228.47 15.76%
Brazil	627,193	240.1	1,634,441	278,835	107.7	6,834,584.54 10.48%
Colombia	117,620	183.3	303,656	56,719	87.6	1,235,853.45 10.68%
Mexico	207,154	141.3	577,487	96,210	66.5	2,872,120.20 8.47%
Panama	8353	158.3	24,610	3770	71.0	99,326.00 10.32%

Source: International Agency for Research on Cancer—All cancers [12,24,27,28,29,30,31].

**Table 2:** Cancer policy landscape in focus countries and inclusion of precision medicine.

Country	National Cancer Policy and Cancer-Related Legislation	Includes Precision/Personalized/Individualized Medicine	Includes Biomarkers	Includes Genetic Markers
Argentina	National Cancer Control Plan 2018–2022 [214]	Yes ^a^	No	No
Brazil	National Policy for the Prevention and Control of Cancer 2023 [215]	No	No	No
	Law No. 14.238 of 19 November 2021—Statute for Persons with Cancer [216] Encompasses objectives aimed at comprehensively addressing the needs of individuals with cancer	No	No	No
	Law No. 12,732 of 22 November 2012 [217] Provides for the treatment of cancer patients	Yes ^a^	No	No
	National Program to Support Oncology Care (2012) [218]	No	No	No
	Strategic Action Plan to Combat Chronic and Non-communicable Diseases (2021–2030) [219]	No	No	No
Colombia	10-Year Cancer Control Plan 2012–2021 [220]	No	No	No
	Law No. 1388 de 2010 [221] Ensures access to early detection and comprehensive treatment services	No	No	No
	Resolution No. 4496 of 2012 [222] Organizes the National Cancer Information System	No	No	No
	Resolution 247 of 2014 [221] Introduces a mandate for all health benefit plan administrators and healthcare institutions to report cancer patients	No	No	No
	Law No. 1384 of 2010 Sandra Ceballos [223] Establishes comprehensive measures for cancer control	No	No	No
	Institutional Public Health Policy for Cancer Control 2021–2023 of the National Institute of Cancerology [224]	No	No	No
Mexico	Specific Action Program for the Prevention and Control of Cancer 2021–2024 [225]	No	No	No
	General Law for the Timely Detection of Cancer in Childhood and Adolescence 2021 [226]	No	No	No
Panama	National Strategic Plan for Cancer Prevention and Control 2019–2029 [227]	No	No	No
	Law 154, 13 May 2020 [228] Creates the national program of support, prevention, and comprehensive care for people suffering from oncological diseases	No	No	No
	Executive Decree 382 2008 [229] Creates the National Commission for the Early Detection of Cancer	No	No	No
	Resolution 291, 16 May 2022 [230] Creates Regional Oncology Units	No	No	No

^a^. Precision medicine is only mentioned, without discussing provisions for its adoption and development.

**Table 4:** Initiatives that aim to ensure equitable access to precision medicine.

Country	Initiative
Sweden	The Genomic Medicine Sweden (GMS) was a bottom-up initiative set in 2017 that gathered a multiprofessional workforce of diagnosticians, clinicians, researchers, and informaticians to coordinate the implementation of genomic-based PM into Swedish healthcare. GMS has established regional Genomic Medicine Centers at all university hospitals across Sweden, stimulating the uptake of genomic-based diagnostics, developed by GMS together with the Swedish research infrastructure Science for Life Laboratory, and supporting equitable access to genomic-based PM across Sweden for patients with rare diseases, cancer, infectious diseases, and complex diseases [141].
England	The NHS Genomic Medicine Service was launched in 2018 and aims to support equitable access to genomics across the NHS in England and provide standardized care across the population, through workforce development, the Genomics Clinical Reference Group, and working with communities and patient groups [279].
US	The All of Us initiative of the NIH recruits participants from diverse backgrounds to improve the makeup of biobanks, considering that nearly all biospecimens used in research come from people of European ancestry. AoU has partnered with Federally Qualified Health Centers, which is a type of community health center whose patient base is comprised largely of people who are uninsured, underinsured, or on Medicaid [280]. In 2016, the US Congress allocated USD 1.5 billion for this program over ten years, subject to an annual appropriations process.

**Table 5:** Suggested solutions to key challenges and gaps identified in the literature.

Challenges and Gaps Identified in the Literature	Suggested Solutions
**Challenges regarding scientific development of precision medicine**	
**Genetic heterogeneity of LatAm’s population**	Recruitment of participants from different backgrounds to improve the makeup of biobanks and the representation of research participants. Advocacy initiatives that use this lack of representation as incentive for the development of precision medicine, but also for research in general, including clinical trials.
**Lack of representation among biomedical research participants**	
**The number of trials related to targeted therapies and molecular profiling in cancer in LatAm is drastically lower than in Europe and the US**	Policies that support fast track and/or referencing of US and/or EMEA to eliminate duplicative efforts and expedite access to novel health technologies (mutual recognition agreements). Standardizing regional legislation and/or establishing a discussion forum with regulatory bodies to share solutions and accelerate the implementation of successful strategies already adopted in specific LatAm countries. Tax incentives and/or technical assistance incentives (regarding approval pathways) for bio/pharmaceutical companies that invest in clinical trials that include diverse subpopulations in LatAm.
**Due to lack of government investment and other alternative support sources, most oncology trials in the region have been sponsored by pharmaceutical companies**	
**The time required for regulatory approval for clinical trial applications in LatAm can limit the interest of pharmaceutical companies in conducting clinical trials in the region**	
**Implementation of biomarkers in clinical practice requires that physicians make many decisions**	[short term] Precision medicine modules included in medical school programs (and related disciplines, incl. lab technicians, pathologists, pharmacists, etc.) and continuous professional development courses to ensure all medics and relevant healthcare workers (i.e., those already practicing and those who are soon to graduate) understand biomarker testing and decision-making models. [medium term] A guidance- and knowledge-sharing platform, including a virtual team of experts and molecular tumor boards (incl. all specialists listed to the left) available from across the globe to help clinics in the early phases of precision medicine implementation to interpret biomarker testing reports, guide decision-making, and share knowledge. [long term] Investment in AI-supported testing report analysis and e-prescribing software (akin to DrugGPT, only geared to address the complexities and nuances of precision medicine) to support interpretation of biomarker testing reports, and to assist clinician decision-making by narrowing down their selection to only the most appropriate treatment regimen for each patient. [long term] Implement a local data repository to capture all instances where the most appropriate treatment was not commenced due to lack of reimbursement or affordability. Use this data as evidence to advocate for broader reimbursement. Invest in research to identify cost-effective application for precision medicine, integrating multi-omics and validating emerging techniques to establish their suitability in LatAm settings.
**Biomarker testing reports must be interpreted by an expert or a team of experts (including medical oncologists, surgical oncologists, pathologists, basic scientists, pharmacists, clinical nurses, physician assistants, and genetic counsellors) to select the optimal treatment to be prescribed**	
**Treatment decisions consider coverage and reimbursement aspects**	
**Challenges implementing precision medicine in healthcare delivery services**	
**Limited availability of adequate infrastructure and trained human resources and laboratories to perform genetic testing and molecular profiling, and to interpret results**	[short term] Precision medicine modules included in medical school programs (and related disciplines, incl. lab technicians, pathologists, pharmacists, etc.) and continuous professional development courses to ensure all medics and relevant healthcare workers (i.e., those already practicing and those who are soon to graduate) understand biomarker testing and decision-making models. [medium term] A guidance- and knowledge-sharing platform, including a virtual team of experts and molecular tumor boards (incl. all specialists listed to the left) available from across the globe to help clinics in the early phases of precision medicine implementation to interpret biomarker testing reports, guide decision-making, and share knowledge. [long term] Investment in AI-supported testing report analysis and e-prescribing software (akin to DrugGPT, only geared to address the complexities and nuances of precision medicine) to support interpretation of biomarker testing reports, and to assist clinician decision-making by narrowing down their selection to only the most appropriate treatment regimen for each patient.
**Requires a multidisciplinary team comprised of clinicians, pathologists, molecular specialists, and others, but such teams are usually unavailable due to the short supply of both financial and human resources**	[short term] Precision medicine modules included in medical school programs (and related disciplines, incl. lab technicians, pathologists, pharmacists, etc.) and continuous professional development courses to ensure all medics and relevant healthcare workers (i.e., those already practicing and those who are soon to graduate) understand biomarker testing and decision-making models. [medium term] A guidance- and knowledge-sharing platform, including a virtual team of experts and molecular tumor boards (incl. all specialists listed to the left) available from across the globe to help clinics in the early phases of precision medicine implementation to interpret biomarker testing reports, guide decision-making, and share knowledge. [long term] Investment in AI-supported testing report analysis and e-prescribing software (akin to DrugGPT, only geared to address the complexities and nuances of precision medicine) to support interpretation of biomarker testing reports, and to assist clinician decision-making by narrowing down their selection to only the most appropriate treatment regimen for each patient.
**Access to trained healthcare professionals, new therapies, and adequate facilities, due to inadequately distributed budget across locations and geographic barriers**	[short term] Precision medicine modules included in medical school programs (and related disciplines, incl. lab technicians, pathologists, pharmacists, etc.) and continuous professional development courses to ensure all medics and relevant healthcare workers (i.e., those already practicing and those who are soon to graduate) understand biomarker testing and decision-making models. [medium term] A guidance- and knowledge-sharing platform, including a virtual team of experts and molecular tumor boards (incl. all specialists listed to the left) available from across the globe to help clinics in the early phases of precision medicine implementation to interpret biomarker testing reports, guide decision-making, and share knowledge. [long term] Investment in AI-supported testing report analysis and e-prescribing software (akin to DrugGPT, only geared to address the complexities and nuances of precision medicine) to support interpretation of biomarker testing reports, and to assist clinician decision-making by narrowing down their selection to only the most appropriate treatment regimen for each patient. [long term] Implement a local data repository to capture all instances where the most appropriate treatment was not commenced due to lack of reimbursement or affordability. Use this data as evidence to advocate for broader reimbursement. A centralized national biomarker center/regional centers to ensure all tests are analyzed and interpreted in a consistent, best practice manner. This would facilitate better patient/treatment matching and more consistent follow-up, monitoring, and research. A national center for precision oncology with representatives/key staff in each region who can travel (or at least provide virtual support) to key areas with unmet need to support clinicians in identifying the most appropriate treatment and providing treatment follow-up. Mobile/virtual precision oncology centers capable of delivering regular (“as regular as possible”—fortnightly or monthly) support to rural and remote clinics implementing precision medicine approaches.
**A lack of stable logistical infrastructure to perform local sequencing and local genomic analysis, and a lack of cost-effective transportation of biological samples to molecular laboratories**	
**Laboratory infrastructure and specialized oncologists are mostly concentrated in urban centers**	
**Insufficient funding for the development of computing, AI, and machine learning to support clinical judgment and managing processes, both of which require improvements in health workforce capacity-building**	[long term] Investment in AI-supported testing report analysis and e-prescribing software (akin to DrugGPT, only geared to address the complexities and nuances of precision medicine) to support interpretation of biomarker testing reports, and to assist clinician decision-making by narrowing down their selection to only the most appropriate treatment regimen for each patient.
**Precision medicine is inherently patient-focused, but its application sometimes hyperfocuses on patient genetics and biomarker reports, rather than reflecting the patient’s values and preferences**	Contribute to existing frameworks (e.g., the PEAR framework) and advocate for their widespread uptake/application to ensure precision medicine is not only patient-focused (customizing treatment based on tissue or receptor types), but also patient-centered (involves and considers patients’ preferences, characteristics, and overall health in treatment plans).
**Available epidemiological cancer data is not reliable, not all countries in the region have population-based cancer registries, and those that do exhibit varying levels of quality and population coverage**	Invest in the development/strengthening of robust cancer registries to accurately assess the cancer burden and guide effective control strategies. Develop a LatAm database/platform where this information on the use of genetic tests, projects, and studies on precision medicine can be collected.
**Challenges regarding economic issues**	
**Limited access to next-generation anti-cancer targeted drugs, mainly due to unaffordability**	Generate legislation that supports access for those who fulfil certain diagnostic and need criteria to access precision medicine (see Ricarte Soto Law in Chile as an example). Empowerment and activation of patient advocacy groups to demand equitable access to precision oncology. Simplify reimbursement processes and address issues of affordability and availability of targeted anti-cancer drugs in the public health system. Improve acquisition mechanisms for cancer drugs that can reduce the overall cost and/or make it more accessible for governments to purchase said drugs.
**Inequities in access to biomarker testing**	
**Healthcare systems in LatAm are characterized by a lack of coverage for populations excluded from social security. Universal healthcare coverage available for only a minority of the LatAm population. In general, many therapeutic options are not reimbursed, leaving patients to face very high out-of-pocket costs**	
**Under-financing of public health systems** **The public investment in clinical research in LatAm is lower than the global average**	Motivate political commitment to address chronic underfunding of public health systems. Prioritize the Primary Health Care approach, e.g., investment of 1% of GDP in primary healthcare.
**Political instability, corruption, and weak governance lead to poor resource allocation, underfunded health systems, and limited prioritization of cancer and precision medicine in public policy agendas**	Foster political commitment by identifying and engaging political leaders able to effect change, and promote the explicit recognition of cancer as a public health priority, backed by the enactment of policies and the corresponding allocation of resources to support their implementation.
**Oncologists hesitate to prescribe targeted therapies because health insurance does not always cover them**	A centralized national biomarker center/regional centers to ensure all tests are analyzed and interpreted in a consistent, best practice manner. This would facilitate better patient/treatment matching and more consistent follow-up, monitoring, and research. Generate legislation that supports access for those who fulfil certain diagnostic and need criteria to access precision medicine (see Ricarte Soto Law in Chile as an example). Empowerment and activation of patient advocacy groups to demand equitable access to precision oncology.
**Not all indicated targeted therapies are commercially available in LatAm, and not all patients can access therapies currently approved**	[short term] Precision medicine modules included in medical school programs (and related disciplines, incl. lab technicians, pathologists, pharmacists, etc.) and continuous professional development courses to ensure all medics and relevant healthcare workers (i.e., those already practicing and those who are soon to graduate) understand biomarker testing and decision-making models. [medium term] A guidance- and knowledge-sharing platform, including a virtual team of experts and molecular tumor boards (incl. all specialists listed to the left) available from across the globe to help clinics in the early phases of precision medicine implementation to interpret biomarker testing reports, guide decision-making, and share knowledge. A centralized national biomarker center/regional centers to ensure all tests are analyzed and interpreted in a consistent, best practice manner. This would facilitate better patient/treatment matching and more consistent follow-up, monitoring, and research. Generate legislation that supports access for those who fulfil certain diagnostic and need criteria to access precision medicine (see Ricarte Soto Law in Chile as an example). Empowerment and activation of patient advocacy groups to demand equitable access to precision oncology.
**Challenges regarding policies and regulations for implementation of precision medicine**	
**At the regional level, international agencies like PAHO lack comprehensive action plans or policies on cancer and precision medicine**	Work with international agencies such as PAHO to develop regional guidelines for comprehensive cancer precision medicine policies and plans.
**Despite all national plans being issued after 2018, there was no mention of biomarkers or genetic markers in any of the national plans, nor in the supporting cancer legislation**	Identify cost-effective applications for precision medicine, integrating multi-omics and validating emerging techniques to establish their efficacy in cancer detection. Include those precision medicine applications deemed appropriate in national settings into existing national cancer plans as they are updated.
**Lack of national policies and laws regarding precision medicine**	Establish a clear “Pathway to Equity” to support equitable access to the national center for precision oncology and centralized national biomarker centers from different starting points, including rural, unprotected areas, and underrepresented populations. As national cancer plans are updated, ensure they include a chapter detailing the current and intended trajectory of precision oncology in the country, alongside the governments’ current and intended investments and priorities on this topic. Clear and comprehensive legal framework to support compliance with laws, regulatory pathways, and patient safety. Collaborative efforts among stakeholders such as scientific societies, governments, payers, healthcare providers, patient groups, research centers, and pharmaceutical companies to consider, inform, and respond to emerging policies, laws, and regulations.
**Delayed approval and registration processes for both broad access and clinical trials**	[short term] Precision medicine modules included in medical school programs (and related disciplines, incl. lab technicians, pathologists, pharmacists, etc.) and continuous professional development courses to ensure all medics and relevant healthcare workers (i.e., those already practicing and those who are soon to graduate) understand biomarker testing and decision-making models. [medium term] A guidance- and knowledge-sharing platform, including a virtual team of experts and molecular tumor boards (incl. all specialists listed to the left) available from across the globe to help clinics in the early phases of precision medicine implementation to interpret biomarker testing reports, guide decision-making, and share knowledge. Generate legislation that supports access for those who fulfil certain diagnostic and need criteria to access precision medicine (see Ricarte Soto Law in Chile as an example). Empowerment and activation of patient advocacy groups to demand equitable access to precision oncology.
**Compliance with law, regulatory pathways, and safety planning is challenging for precision therapies and diagnostics in LatAm and contributes to regulatory lags**	[short term] Precision medicine modules included in medical school programs (and related disciplines, incl. lab technicians, pathologists, pharmacists, etc.) and continuous professional development courses to ensure all medics and relevant healthcare workers (i.e., those already practicing and those who are soon to graduate) understand biomarker testing and decision-making models. [medium term] A guidance- and knowledge-sharing platform, including a virtual team of experts and molecular tumor boards (incl. all specialists listed to the left) available from across the globe to help clinics in the early phases of precision medicine implementation to interpret biomarker testing reports, guide decision-making, and share knowledge. Simplify, clarify, and communicate precision therapies and diagnostics regulations in LatAm to ensure all necessary stakeholders have up-to-date information and understanding required to abide by them.
**Regulatory processes are independent, but HTA evaluations may depend on the healthcare system’s capacity to adopt and reimburse new drugs**	Align HTA evaluations with regulatory processes, adopting value-based pricing to streamline drug adoption while balancing innovation and affordability.

## References

[1] Mount Sinai (2024). Targeted Therapies for Cancer. https://www.mountsinai.org/health-library/special-topic/targeted-therapies-for-cancer.

[2] National Library of Medicine (2022). What Is Precision Medicine?. https://medlineplus.gov/genetics/understanding/precisionmedicine/definition/.

[3] National Cancer Institute (2022). Biomarker Testing. https://www.cancer.gov/publications/dictionaries/cancer-terms/def/biomarker-testing.

[4] National Cancer Institute (2022). Targeted Therapy to Treat Cancer. https://www.cancer.gov/about-cancer/treatment/types/targeted-therapies.

[5] World Health Organization (2023). Health Technologies. https://www.who.int/europe/news-room/fact-sheets/item/health-technologies.

[6] Mathur S., Sutton J. (2017). Personalized medicine could transform healthcare. Biomed. Rep..

[7] CSDH (2008). Closing the Gap in a Generation: Health Equity Through Action on the Social Determinants of Health. Final Report of the Commission on Social Determinants of Health. Geneva. https://iris.who.int/bitstream/handle/10665/43943/9789241563703_eng.pdf.

[8] World Health Organization (2024). Health Equity. https://www.who.int/health-topics/health-equity.

[9] Nature Medicine (2021). Precision medicine needs an equity agenda. Nat. Med..

[10] Khoury M.J., Bowen S., Dotson W.D., Drzymalla E., Green R.F., Goldstein R., Kolor K., Liburd L.C., Spearling L.S., Bunnell R. (2022). Health equity in the implementation of genomics and precision medicine: A public health imperative. Genet. Med..

[11] International Agency for Research on Cancer (2024). Global Cancer Observatory—United States of America. Cancer Today. [Fact Sheet]. https://gco.iarc.who.int/media/globocan/factsheets/populations/840-united-states-of-america-fact-sheet.pdf.

[12] International Agency for Research on Cancer (2024). Global Cancer Observatory—World. Cancer Today. [Fact Sheet]. https://gco.iarc.who.int/media/globocan/factsheets/populations/900-world-fact-sheet.pdf.

[13] López-Cortés A., Guerrero S., Redal M., Alvarado A., Quiñones L. (2017). State of Art of Cancer Pharmacogenomics in Latin American Populations. Int. J. Mol. Sci..

[14] Ruiz R., Strasser-Weippl K., Touya D., Herrero Vincent C., Hernandez-Blanquisett A., St Louis J., Bukowski A., Goss P.E. (2017). Improving access to high-cost cancer drugs in Latin America: Much to be done. Cancer.

[15] Organization for Economic Co-Operation and Development (2023). Health at a Glance: Latin America and the Caribbean 2023.

[16] World Health Organization (2022). Cancer Fact Sheet. https://www.who.int/news-room/fact-sheets/detail/cancer.

[17] Piñeros M., Laversanne M., Barrios E., de Camargo Cancela M., de Vries E., Pardo C., Bray F. (2022). An updated profile of the cancer burden, patterns and trends in Latin America and the Caribbean. Lancet Reg. Health Am..

[18] Sarfati D. (2019). Why Social Inequalities Matter in the Cancer Continuum.

[19] Espina C., Feliu A., Maza M., Almonte M., Ferreccio C., Finck C., Herrero F., Dommarco J.R., Almeida L.M., Arrosi S. (2023). Latin America and the Caribbean Code Against Cancer 1st Edition: 17 cancer prevention recommendations to the public and to policy-makers (World Code Against Cancer Framework). Cancer Epidemiol..

[20] Social Development Division of the Economic Commission for Latin America and the Caribbean (2023). Social Panorama of Latin America and the Caribbean 2023: Labour Inclusion as a Key Axis of Inclusive Social Development. https://hdl.handle.net/11362/68703.

[21] World Bank (2024). Poverty Headcount Ratio at National Poverty Lines (% of Population)—Argentina, Brazil, Colombia, Mexico, Panama. https://data.worldbank.org/indicator/SI.POV.NAHC?locations=AR-BR-CO-MX-PA.

[22] Agência IBGE (2023). Poverty Drops to 31.6% of the Population in 2022, After Reaching 36.7% in 2021. https://agenciadenoticias.ibge.gov.br/en/agencia-news/2184-news-agency/news/38574-poverty-drops-to-31-6-of-the-population-in-2022-after-reaching-36-7-in-2021.

[23] Global Burden of Disease Collaborative Network (2020). Global Burden of Disease Study 2019 (GBD 2019) Results. [Database]. Institute for Health Metrics and Evaluation (IHME). https://vizhub.healthdata.org/gbd-results/.

[24] International Agency for Research on Cancer (2024). Global Cancer Observatory—Latin America and the Caribbean. Cancer Today. [Fact Sheet]. https://gco.iarc.who.int/media/globocan/factsheets/populations/904-latin-america-and-the-caribbean-fact-sheet.pdf.

[25] United Nations (2022). Revision of World Population Prospects. Department of Economic and Social Affairs. Population Division. https://population.un.org/wpp/.

[26] World Health Organization Cancer Tomorrow: Estimated Number of New Cases from 2022 to 2050, Incidence, Both Sexes, Age [0–85+]. https://gco.iarc.who.int/tomorrow/en/dataviz/tables?mode=population&years=2050.

[27] International Agency for Research on Cancer (2024). Global Cancer Observatory—Argentina. Cancer Today. [Fact Sheet]. https://gco.iarc.who.int/media/globocan/factsheets/populations/32-argentina-fact-sheet.pdf.

[28] International Agency for Research on Cancer (2024). Global Cancer Observatory—Brazil. Cancer Today. [Fact Sheet]. https://gco.iarc.who.int/media/globocan/factsheets/populations/76-brazil-fact-sheet.pdf.

[29] International Agency for Research on Cancer (2024). Global Cancer Observatory—Colombia. Cancer Today. [Fact Sheet]. https://gco.iarc.who.int/media/globocan/factsheets/populations/170-colombia-fact-sheet.pdf.

[30] International Agency for Research on Cancer (2024). Global Cancer Observatory—Mexico. Cancer Today. [Fact Sheet]. https://gco.iarc.who.int/media/globocan/factsheets/populations/484-mexico-fact-sheet.pdf.

[31] International Agency for Research on Cancer (2024). Global Cancer Observatory—Panama. Cancer Today. [Fact Sheet]. https://gco.iarc.who.int/media/globocan/factsheets/populations/591-panama-fact-sheet.pdf.

[32] Barrios C.H., Werutsky G., Mohar A., Ferrigno A.S., Müller B.G., Bychkovsky B.L., Castro E.C.J., Uribe C.J., Villarreal-Garza C., Soto-Perez-de-Celis E. (2021). Cancer control in Latin America and the Caribbean: Recent advances and opportunities to move forward. Lancet Oncol..

[33] Prager G.W., Braga S., Bystricky B., Qvortrup C., Criscitiello C., Esin E. (2018). Global cancer control: Responding to the growing burden, rising costs and inequalities in access. ESMO Open.

[34] Worldometer (2024). GDP per Capita. https://www.worldometers.info/gdp/gdp-per-capita/.

[35] Ries L., Trama A., Nakata K., Gatta G., Botta L., Bleyer A. (2017). Cancer Incidence, Survival, and Mortality Among Adolescents and Young Adults.

[36] Gupta S., Harper A., Ruan Y., Barr R., Frazier A.L., Ferlay J., Steliarova-Foucher E., Fidler-Benaoudia M.M. (2020). International Trends in the Incidence of Cancer Among Adolescents and Young Adults. JNCI J. Natl. Cancer Inst..

[37] Rice D.P. (2000). Cost of illness studies: What is good about them?. Inj. Prev..

[38] Marmot M., Wilkinson R., Marmot M., Wilkinson R. (2005). Social Determinants of Health.

[39] Chen S., Cao Z., Prettner K., Kuhn M., Yang J., Jiao L., Wang Z., Li W., Geldsetzer P., Bärnighausen T. (2023). Estimates and Projections of the Global Economic Cost of 29 Cancers in 204 Countries and Territories From 2020 to 2050. JAMA Oncol..

[40] de la Torre-Luque A., Gambara H., López E., Cruzado J.A. (2016). Psychological treatments to improve quality of life in cancer contexts: A meta-analysis. Int. J. Clin. Health Psychol..

[41] Finck C., Barradas S., Zenger M., Hinz A. (2018). Quality of life in breast cancer patients: Associations with optimism and social support. Int. J. Clin. Health Psychol..

[42] Chaves-Cardona R., Romero-Prada M., Ocampo M.V., Gallo D., Gómez L.M., Clavijo N. (2021). Utility and health-related quality of life measures in adult Colombian patients with solid tumours. Ecancermedicalscience.

[43] Santos MN dos, Brito RG de (2022). Qualidade de vida em pacientes com diagnóstico de câncer no Brasil: Uma revisão sistemática. Res. Soc. Dev..

[44] Mejía-Rojas M.E., Contreras-Rengifo A., Hernández-Carrillo M. (2020). Calidad de vida en mujeres con cáncer de mama sometidas a quimioterapia en Cali, Colombia. Biomédica.

[45] Gonzalez L., Bardach A., Palacios A., Peckaitis C., Ciapponi A., Pichón-Riviere A., Augustovski F. (2021). Health-Related Quality of Life in Patients with Breast Cancer in Latin America and the Caribbean: A Systematic Review and Meta-Analysis. Oncologist.

[46] Sierra-Guerra K.L., Viveros-Contreras C., Martínez-Carrillo G., Hernández-León O., Caballero-Ambriz G. (2014). Calidad de vida en pacientes con cáncer de próstata, operados de prostatectomía radical laparoscópica. Rev. Mex. Urol..

[47] Acevedo-Ibarra J.N., Juárez-García D.M., Espinoza-Velazco A., Buenaventura-Cisneros S. (2021). Quality of life in Mexican colorectal cancer patients: Analysis with sociodemographic, medical, and psychological variables. Psychol Health Med..

[48] Guerra-Martín M.D., Casado-Espinosa M.D.R., Gavira-López Y., Holgado-Castro C., López-Latorre I., Borrallo-Riego Á. (2023). Quality of Life in Caregivers of Cancer Patients: A Literature Review. Int. J. Environ. Res. Public Health.

[49] Molassiotis A., Wang M. (2022). Understanding and Supporting Informal Cancer Caregivers. Curr. Treat. Options Oncol..

[50] Espinola N., Pichon-Riviere A., Casarini A., Alcaraz A., Bardach A., Williams C., Cairoli F.R., Augustovski F., Palacios A. (2023). Making visible the cost of informal caregivers’ time in Latin America: A case study for major cardiovascular, cancer and respiratory diseases in eight countries. BMC Public Health.

[51] Xiang E., Guzman P., Mims M., Badr H. (2022). Balancing Work and Cancer Care: Challenges Faced by Employed Informal Caregivers. Cancers.

[52] United Nations Economic Commission for Europe (2019). Supporting Informal Carers—Six Policy Challenges and How to Meet Them. https://unece.org/population/news/supporting-informal-carers-six-policy-challenges-and-how-meet-them.

[53] World Health Organization (2020). WHO Report on Cancer: Setting Priorities, Investing Wisely and Providing Care for All. https://www.who.int/publications/i/item/9789240001299.

[54] Llera A.S. (2023). A fresh perspective on Latin America cancer care: Uncovering hidden messages in unconventional data sources. Lancet Reg. Health Am..

[55] de Lemos L.L.P., Carvalho de Souza M., Pena Moreira D., Ribeiro Fernandes Almeida P.H., Godman B., Verguet S., Junior A.A.G., Cherchiglia M.L. (2019). Stage at diagnosis and stage-specific survival of breast cancer in Latin America and the Caribbean: A systematic review and meta-analysis. PLoS ONE.

[56] Carioli G., Bertuccio P., Malvezzi M., Rodriguez T., Levi F., Boffetta P., La Vecchia C., Negri E. (2020). Cancer mortality predictions for 2019 in Latin America. Int. J. Cancer.

[57] Brand N.R., Qu L.G., Chao A., Ilbawi A.M. (2019). Delays and Barriers to Cancer Care in Low- and Middle-Income Countries: A Systematic Review. Oncologist.

[58] Unger-Saldaña K. (2014). Challenges to the early diagnosis and treatment of breast cancer in developing countries. World J. Clin. Oncol..

[59] Werutsky G., Barrios C.H., Cardona A.F., Albergaria A., Valencia A., Ferreira C.G. (2021). Perspectives on emerging technologies, personalised medicine, and clinical research for cancer control in Latin America and the Caribbean. Lancet Oncol..

[60] Cazap E. (2018). Breast Cancer in Latin America: A Map of the Disease in the Region. Am. Soc. Clin. Oncol. Educ. Book.

[61] Rosa D.D., Bines J., Werutsky G., Barrios C.H., Cronemberger E., Queiroz G.S., Lima V.C.C., Freitas-Júnior R., Couto J.O., Emerenciano K. (2020). The impact of sociodemographic factors and health insurance coverage in the diagnosis and clinicopathological characteristics of breast cancer in Brazil: AMAZONA III study (GBECAM 0115). Breast Cancer Res. Treat..

[62] Martinez-Cannon B.A., Zertuche-Maldonado T., de la Rosa Pacheco S., Cardona-Huerta S., Canavati-Marcos M., Gomez-Macias G.S., Villarreal-Garza C. (2020). Comparison of characteristics in Mexican women with breast cancer according to healthcare coverage. Women’s Health.

[63] Cordeiro de Lima V.C., Gelatti A., Moura J.F.P., Fares A.F., de Castro G., Mathias C., Terra R.M., Werustky G., Corassa M., Araújo L.H.L. (2024). Health Services Access Inequalities in Brazil Result in Poorer Outcomes for Stage III NSCLC—RELANCE/LACOG 0118. JTO Clin. Res. Rep..

[64] Nuche-Berenguer B., Sakellariou D. (2019). Socioeconomic determinants of cancer screening utilisation in Latin America: A systematic review. PLoS ONE.

[65] Ruiz de Castilla E.M., Mayrides M., González H., Vidangossy F., Corbeaux T., Ortiz N. (2024). Implementing precision oncology in Latin America to improve patient outcomes: The status quo and a call to action for key stakeholders and decision-makers. Ecancermedicalscience.

[66] Moye-Holz D., Soria Saucedo R., van Dijk J.P., Reijneveld S.A., Hogerzeil H.V. (2018). Access to innovative cancer medicines in a middle-income country—The case of Mexico. J. Pharm. Policy Pract..

[67] Vargas-Pelaez C.M., Rover M.R.M., Soares L., Blatt C.R., Mantel-Teeuwisse A.K., Rossi F.A., Restrepo L.G., Latorre M.C., López J.J., Bürgin M.T. (2019). Judicialization of access to medicines in four Latin American countries: A comparative qualitative analysis. Int. J. Equity Health.

[68] Chagas V.O., Provin M.P., Mota P.A.P., Guimarães R.A., Amaral R.G. (2020). Institutional strategies as a mechanism to rationalize the negative effects of the judicialization of access to medicine in Brazil. BMC Health Serv. Res..

[69] Andia T.S., Lamprea E. (2019). Is the judicialization of health care bad for equity? A scoping review. Int. J. Equity Health.

[70] Salha L.A., Reis F.C., Gonçalves R.M., Lima JHda S., Salha N.A., Pinto R.P., Menezes J.E., Oliveira E.P., Ferreira P.L., Barbosa M.A. (2022). Judicialization of health: Profile of demands for oncological medicines in a state in the central region of Brazil. Int. J. Equity Health.

[71] Biehl J., Socal MP, Amon, J (2016). J. The Judicialization of Health and the Quest for State Accountability: Evidence from 1262 Lawsuits for Access to Medicines in Southern Brazil. Health Hum. Rights.

[72] Civil Society Engagement Mechanism (2019). Why the 2019 UN High-Level Meeting on Universal Health Coverage Should Encourage All Countries to Achieve This Target. https://csemonline.net/wp-content/uploads/2019/07/WHY-5-of-GDP-1.pdf.

[73] Our World in Data (2024). Government Health Expenditure as a Share of GDP, 1880 to 2021. https://ourworldindata.org/grapher/public-health-expenditure-share-gdp.

[74] Piñeros M., Abriata M.G., de Vries E., Barrios E., Bravo L.E., Cueva P., Cancela M.C., Fernández L., Gil E., Luciani S. (2021). Progress, challenges and ways forward supporting cancer surveillance in Latin America. Int. J. Cancer.

[75] World Health Organization (2024). Health Inequality Monitor- About WHO’s Work on Health Inequality Monitoring. https://www.who.int/data/inequality-monitor/about.

[76] Aday L.A., Fleming G.V., Andersen R. (1984). Access to Medical Care in the U.S.: Who Has It, Who Doesn’t.

[77] Whitehead M. (1992). The Concepts and Principles of Equity and Health. Int. J. Health Serv..

[78] Lee H., Kim D., Lee S., Fawcett J. (2020). The concepts of health inequality, disparities and equity in the era of population health. Appl. Nurs. Res..

[79] Arcaya M.C., Arcaya A.L., Subramanian S.V. (2015). Inequalities in health: Definitions, concepts, and theories. Glob. Health Action.

[80] Pan American Health Organization (1999). Methodological summaries: Measuring inequity in health. Epidemiol. Bull..

[81] Yao Q., Li X., Luo F., Yang L., Liu C., Sun J. (2019). The historical roots and seminal research on health equity: A referenced publication year spectroscopy (RPYS) analysis. Int. J. Equity Health.

[82] World Health Organization (1948). Constitution of the World Health Organization. https://www.who.int/about/governance/constitution.

[83] UN General Assembly (1948). Universal Declaration of Human Rights|United Nations. General Assembly Resolution. https://documents.un.org/doc/resolution/gen/nr0/043/88/pdf/nr004388.pdf.

[84] World Health Organization (1978). Report of the International Conference on Primary Health Care, Alma-Ata, USSR, 6–12 September 1978. https://iris.who.int/bitstream/handle/10665/39228/9241800011.pdf?sequence=1.

[85] World Health Organization (2015). Health in 2015: From MDGs, Millennium Development Goals to SDGs, Sustainable Development Goals. https://iris.who.int/handle/10665/200009.

[86] Braveman P., Arkin E., Orleans T., Proctor D., Acker J., Plough A. (2018). What is Health Equity?. Behav. Sci. Policy.

[87] Aldrighetti C.M., Niemierko A., Van Allen E., Willers H., Kamran S.C. (2021). Racial and Ethnic Disparities Among Participants in Precision Oncology Clinical Studies. JAMA Netw. Open.

[88] Edwards T.L., Breeyear J., Piekos J.A., Velez Edwards D.R. (2020). Equity in Health: Consideration of Race and Ethnicity in Precision Medicine. Trends Genet..

[89] Kavanagh M.M., Norato L.F., Friedman E.A., Armbrister A.N. (2021). Planning for health equity in the Americas: An analysis of national health plans. Rev. Panam. Salud Pública.

[90] Ruano A.L., Rodríguez D., Rossi P.G., Maceira D. (2021). Understanding inequities in health and health systems in Latin America and the Caribbean: A thematic series. Int. J. Equity Health.

[91] Centers for Disease Control and Prevention (2023). Equity in Cancer Prevention and Control. https://www.cdc.gov/cancer/health-equity/equity.htm.

[92] Kale S., Hirani S., Vardhan S., Mishra A., Ghode D.B., Prasad R., Wanjari M. (2023). Addressing Cancer Disparities Through Community Engagement: Lessons and Best Practices. Cureus.

[93] National Cancer Institute (2024). Cancer Disparities. https://www.cancer.gov/about-cancer/understanding/disparities.

[94] World Health Organization Improving Service Access and Quality. https://www.who.int/activities/improving-service-access-and-quality.

[95] World Health Organization (2023). Universal Health Coverage. Report by the Director-General. 154th Session. https://apps.who.int/gb/ebwha/pdf_files/EB154/B154_6-en.pdf.

[96] World Health Organization (2020). Retention of the Health Workforce in Rural and Remote Areas: A Systematic Review. https://iris.who.int/bitstream/handle/10665/337300/9789240013865-eng.pdf?sequence=1.

[97] Thomas S., Sagan A., Larkin J., Cylus J., Figueiras J., Karanikolos M. (2020). Strengthening Health Systems Resilience: Key Concepts and Strategies.

[98] Kieny M.P., Bekedam H., Dovlo D., Fitzgerald J., Habicht J., Harrison G., Kluge H., Lin V., Menabde N., Mirza Z. (2017). Strengthening health systems for universal health coverage and sustainable development. Bull. World Health Organ..

[99] World Health Organization (2016). Patient Engagement: Technical Series on Safer Primary Care. https://iris.who.int/bitstream/handle/10665/252269/9789241511629-eng.pdf?sequence=1.

[100] Handtke O., Schilgen B., Mösko M. (2019). Culturally competent healthcare—A scoping review of strategies implemented in healthcare organizations and a model of culturally competent healthcare provision. PLoS ONE.

[101] Alvarado-Cabrero I., Doimi F., Ortega V., de Oliveira Lima J.T., Torres R., Torregrosa L. (2021). Recommendations for streamlining precision medicine in breast cancer care in Latin America. Cancer Rep..

[102] Gilardino R.E., Valanzasca P., Rifkin S.B. (2022). Has Latin America achieved universal health coverage yet? Lessons from four countries. Arch. Public Health.

[103] Pan American Health Organization (2024). Universal Health- On the Road to Universal Health for Everyone, Everywhere. https://www.paho.org/en/universal-health.

[104] Abramovich V., Pautassi L. (2008). Judicial activism in the Argentine health system: Recent trends. Health Hum. Rights.

[105] Filho L.B. (2022). The Right to Health as a Tool of Social Control: Compulsory Treatment Orders by Courts in Brazil. Health Hum. Rights.

[106] Rosa R.M., Alberto I.C. (2004). Universal health care for Colombians 10 years after Law 100: Challenges and opportunities. Health Policy.

[107] Block M.A.G., Morales H.R., Hurtado L.C., Balandrán A., Méndez E. (2020). Mexico- Health System Review. https://iris.who.int/handle/10665/334334.

[108] Pan American Health Organization (2007). Health Systems Profile Panama: Monitoring and Analyzing Health Systems Change. https://www3.paho.org/hq/dmdocuments/2010/Health_System_Profile-Panama_2008.pdf.

[109] Arai R.J., Guindalini R.S.C., Llera A.S., O’Connor J.M., Muller B., Lema M., Freitas H.C., Soria T., Delgado L., Landaverde D. (2019). Personalizing Precision Oncology Clinical Trials in Latin America: An Expert Panel on Challenges and Opportunities. Oncologist.

[110] Sussman L., Garcia-Robledo J.E., Ordóñez-Reyes C., Forero Y., Mosquera A.F. (2022). Ruíz-Patiño, A.; Chamorro, D.F.; Cardona, A.F. Integration of artificial intelligence and precision oncology in Latin America. Front. Med. Technol..

[111] Penchansky R., Thomas J.W. (1981). The Concept of Access. Med. Care.

[112] Azeredo-da-Silva A.F., Zanotto B.S., Martins F., Navarro N., Alencar R., Medeiros C. (2024). Health care accessibility and mobility in breast cancer: A Latin American perspective. BMC Health Serv. Res..

[113] Malek Pascha V.A., Sun L., Gilardino R., Legood R. (2021). Telemammography for breast cancer screening: A cost-effective approach in Argentina. BMJ Health Care Inform..

[114] Bogdanova A., Andrawos C., Constantinou C. (2022). Cervical cancer, geographical inequalities, prevention and barriers in resource depleted countries (Review). Oncol. Lett..

[115] Patel M.I., Lopez A.M., Blackstock W., Reeder-Hayes K., Moushey E.A., Phillips J., Tap W. (2020). Cancer Disparities and Health Equity: A Policy Statement From the American Society of Clinical Oncology. J. Clin. Oncol..

[116] Marmot M. (2018). Health equity, cancer, and social determinants of health. Lancet Glob. Health.

[117] National Human Genome Research Institute (2024). Precision Medicine. https://www.genome.gov/genetics-glossary/Precision-Medicine.

[118] Nature Mental Health (2023). The right treatment for each patient: Unlocking the potential of personalized psychiatry. Nat. Ment. Health.

[119] Jalilian L., Cannesson M. (2020). Precision medicine in anesthesiology. Int. Anesth. Clin..

[120] Fernandes B.S., Williams L.M., Steiner J., Leboyer M., Carvalho A.F., Berk M. (2017). The new field of ‘precision psychiatry’. BMC Med..

[121] Konstantinidou M.K., Karaglani M., Panagopoulou M., Fiska A., Chatzaki E. (2017). Are the Origins of Precision Medicine Found in the Corpus Hippocraticum?. Mol. Diagn. Ther..

[122] Institute of Medicine (2001). Committee on Quality of Health Care in America. Crossing the Quality Chasm.

[123] Watanabe J.H., Tarn D.M., Hirsch J.D. (2021). Evolution of Precision Medicine: Applying a Population-Based Evidence Assessment Repository to Achieve Patient-Centered Outcomes at the Point of Care. Value Outcomes Spotlight.

[124] National Human Genome Research Institute (2024). The Human Genome Project. https://www.genome.gov/human-genome-project.

[125] Koboldt D.C., Steinberg K.M., Larson D.E., Wilson R.K., Mardis E.R. (2013). The Next-Generation Sequencing Revolution and Its Impact on Genomics. Cell.

[126] Brittain H.K., Scott R., Thomas E. (2017). The rise of the genome and personalised medicine. Clin. Med..

[127] The White House (2015). President Obama’s Precision Medicine Initiative. https://obamawhitehouse.archives.gov/the-press-office/2015/01/30/fact-sheet-president-obama-s-precision-medicine-initiative.

[128] US Food and Drug Administration (2018). Precision Medicine. https://www.fda.gov/medical-devices/in-vitro-diagnostics/precision-medicine.

[129] Baird A., Westphalen C.B., Blum S., Nafria B., Knott T., Sargeant I., Harnik H., Brooke N., Wicki N., Wong-Rieger D. (2023). How can we deliver on the promise of precision medicine in oncology and beyond? A practical roadmap for action. Health Sci. Rep..

[130] Khoury M.J., Holt K.E. (2021). The impact of genomics on precision public health: Beyond the pandemic. Genome Med..

[131] Tebani A., Afonso C., Marret S., Bekri S. (2016). Omics-Based Strategies in Precision Medicine: Toward a Paradigm Shift in Inborn Errors of Metabolism Investigations. Int. J. Mol. Sci..

[132] Fox B.I., Felkey B.G. (2016). Precision Medicine: Considerable Potential Combined with Important Challenges. Hosp. Pharm..

[133] Carlsten C., Brauer M., Brinkman F., Brook J., Daley D., McNagny K., Pui M., Royce D., Takaro T., Denburg J. (2014). Genes, the environment and personalized medicine. EMBO Rep..

[134] McGrath S., Ghersi D. (2016). Building towards precision medicine: Empowering medical professionals for the next revolution. BMC Med. Genom..

[135] Johnson K.B., Wei W., Weeraratne D., Frisse M.E., Misulis K., Rhee K., Zhao J., Snowdon J.L. (2021). Precision Medicine, AI, and the Future of Personalized Health Care. Clin. Transl. Sci..

[136] Giordano C., Brennan M., Mohamed B., Rashidi P., Modave F., Tighe P. (2021). Accessing Artificial Intelligence for Clinical Decision-Making. Front. Digit. Health.

[137] Hassan M., Awan F.M., Naz A., deAndrés-Galiana E.J., Alvarez O., Cernea A., Fernández-Brillet L., Fernández-Martínez J.L., Kloczkowski A. (2022). Innovations in Genomics and Big Data Analytics for Personalized Medicine and Health Care: A Review. Int. J. Mol. Sci..

[138] Kalra D. (2019). The Importance of Real-World Data to Precision Medicine. Per. Med..

[139] Lamichhane P., Agrawal A. (2023). Precision medicine and implications in medical education. Ann. Med. Surg..

[140] Panduro A., Roman S. (2020). Personalized Medicine in Latin America. Per. Med..

[141] Stenzinger A., Moltzen E.K., Winkler E., Molnar-Gabor F., Malek N., Costescu A., Jensen B.N., Nowak F., Pinto C., Ottersen O.P. (2023). Implementation of precision medicine in healthcare—A European perspective. J. Intern. Med..

[142] Argotti U., Leyens L., Lisbona C., López P., Alonso-Orgaz S., Nevado A., Cozzi V. (2023). Comparison of the Latin America Regulation Landscape and International Reference Health Authorities to Hasten Drug Registration and Clinical Research Applications. Ther. Innov. Regul. Sci..

[143] Slikker W. (2018). Biomarkers and their impact on precision medicine. Exp. Biol. Med..

[144] World Health Organization (1993). International Programme on Chemical Safety Biomarkers and Risk Assessment: Concepts and Principles. www.inchem.org/documents/ehc/ehc/ehc155.htm.

[145] Food and Drug Administration (2021). About Biomarkers and Qualification. https://www.fda.gov/drugs/biomarker-qualification-program/about-biomarkers-and-qualification.

[146] Califf R.M. (2018). Biomarker definitions and their applications. Exp. Biol. Med..

[147] Shibata M., Hoque M.O. (2018). Development of biomarkers for real precision medicine. Transl. Lung Cancer Res..

[148] Perez E.A. (2020). Biomarkers and Precision Medicine in Oncology Practice and Clinical Trials. Advancing the Science of Cancer in Latinos.

[149] Orsini A., Diquigiovanni C., Bonora E. (2023). Omics Technologies Improving Breast Cancer Research and Diagnostics. Int. J. Mol. Sci..

[150] Liao J., Li X., Gan Y., Han S., Rong P., Wang W., Li W., Zhou L. (2023). Artificial intelligence assists precision medicine in cancer treatment. Front. Oncol..

[151] Bhinder B., Gilvary C., Madhukar N.S., Elemento O. (2021). Artificial Intelligence in Cancer Research and Precision Medicine. Cancer Discov..

[152] Stein M.K., Oluoha O., Patel K., VanderWalde A. (2021). Precision Medicine in Oncology: A Review of Multi-Tumor Actionable Molecular Targets with an Emphasis on Non-Small Cell Lung Cancer. J. Pers. Med..

[153] The Lancet (2021). 20 years of precision medicine in oncology. Lancet.

[154] Vargas A.J., Harris C.C. (2016). Biomarker development in the precision medicine era: Lung cancer as a case study. Nat. Rev. Cancer.

[155] Personalized Medicine Coalition (2018). Personalized Medicine at FDA: A Progress & Outlook Report. https://www.personalizedmedicinecoalition.org/Userfiles/PMC-Corporate/file/PM_at_FDA_A_Progress_and_Outlook_Report.pdf.

[156] Murciano-Goroff Y.R., Taylor B.S., Hyman D.M., Schram A.M. (2020). Toward a More Precise Future for Oncology. Cancer Cell..

[157] Tsimberidou A.M., Fountzilas E., Nikanjam M., Kurzrock R. (2020). Review of precision cancer medicine: Evolution of the treatment paradigm. Cancer Treat. Rev..

[158] Bhat A.H., Khaja U.M., Ahmed M., Khan W.Y., Ganie S.A. (2023). Pharmacogenomics in cancer. Pharmacogenomics.

[159] Drugan T., Leucuța D. (2024). Evaluating Novel Biomarkers for Personalized Medicine. Diagnostics.

[160] Jørgensen J.T., Westergaard N. (2022). Predictive biomarkers and personalized pharmacotherapy. Expert. Rev. Mol. Diagn..

[161] Das S., Dey M.K., Devireddy R., Gartia M.R. (2023). Biomarkers in Cancer Detection, Diagnosis, and Prognosis. Sensors.

[162] Sheng K.L., Kang L., Pridham K.J., Dunkenberger L.E., Sheng Z., Varghese R.T. (2020). An integrated approach to biomarker discovery reveals gene signatures highly predictive of cancer progression. Sci. Rep..

[163] Al-Tashi Q., Saad M.B., Muneer A., Qureshi R., Mirjalili S., Sheshadri A., Le X., Vokes N.I., Zhang J., Wu J. (2023). Machine Learning Models for the Identification of Prognostic and Predictive Cancer Biomarkers: A Systematic Review. Int. J. Mol. Sci..

[164] Zhou Y., Peng S., Wang H., Cai X., Wang Q. (2024). Review of Personalized Medicine and Pharmacogenomics of Anti-Cancer Compounds and Natural Products. Genes.

[165] Chen J., Sun M., Shen B. (2015). Deciphering oncogenic drivers: From single genes to integrated pathways. Brief. Bioinform..

[166] US Food and Drug Administration (2024). Table of Pharmacogenomic Biomarkers in Drug Labeling. https://www.fda.gov/drugs/science-and-research-drugs/table-pharmacogenomic-biomarkers-drug-labeling.

[167] Zhou Y., Tao L., Qiu J., Xu J., Yang X., Zhang Y., Tian X., Guan X., Cen X., Zhao Y. (2024). Tumor biomarkers for diagnosis, prognosis and targeted therapy. Signal Transduct. Target. Ther..

[168] Dienstmann R. (2021). WS04.02 Access to Biomarker Testing in Latin America. J. Thorac. Oncol..

[169] Calderon-Aparicio A., Orue A. (2019). Precision oncology in Latin America: Current situation, challenges and perspectives. Ecancermedicalscience.

[170] Schienda J., Stopfer J. (2020). Cancer Genetic Counseling—Current Practice and Future Challenges. Cold Spring Harb. Perspect. Med..

[171] Mosele F., Remon J., Mateo J., Westphalen C.B., Barlesi F., Lolkema M.P., Normanno N., Scarpa A., Robson M., Meric-Bernstam F. (2020). Recommendations for the use of next-generation sequencing (NGS) for patients with metastatic cancers: A report from the ESMO Precision Medicine Working Group. Ann. Oncol..

[172] Gavan S.P., Thompson A.J., Payne K. (2018). The economic case for precision medicine. Expert. Rev. Precis. Med. Drug Dev..

[173] Quinn R., Patel R., Sison C., Singh A., Zhu X.H. (2021). Impact of Precision Medicine on Clinical Outcomes: A Single-Institution Retrospective Study. Front. Oncol..

[174] Anderson E.C., DiPalazzo J., Lucas F.L., Hall M.J., Antov A., Helbig P., Bourne J., Graham L., Gaitor L., Lu-Emerson C. (2024). Genome-matched treatments and patient outcomes in the Maine Cancer Genomics Initiative (MCGI). NPJ Precis. Oncol..

[175] Haslem D.S., Chakravarty I., Fulde G., Gilbert H., Tudor B.P., Lin K., Ford J.M., Nadauld L.D. (2018). Precision oncology in advanced cancer patients improves overall survival with lower weekly healthcare costs. Oncotarget.

[176] Kato S., Kim K.H., Lim H.J., Boichard A., Nikanjam M., Weihe E. (2020). Real-world data from a molecular tumor board demonstrates improved outcomes with a precision N-of-One strategy. Nat. Commun..

[177] Song I.W., Vo H.H., Chen Y.S., Baysal M.A., Kahle M., Johnson A., Tsimberidou A.M. (2023). Precision Oncology: Evolving Clinical Trials across Tumor Types. Cancers.

[178] Rulten S.L., Grose R.P., Gatz S.A., Jones J.L., Cameron A.J.M. (2023). The Future of Precision Oncology. Int. J. Mol. Sci..

[179] Senft D., Leiserson M.D.M., Ruppin E., Ronai Z.A. (2017). Precision Oncology: The Road Ahead. Trends Mol. Med..

[180] Pich O., Bailey C., Watkins T.B.K., Zaccaria S., Jamal-Hanjani M., Swanton C. (2022). The translational challenges of precision oncology. Cancer Cell.

[181] National Cancer Institute (2022). Immune Checkpoint Inhibitor. https://www.cancer.gov/about-cancer/treatment/types/immunotherapy/checkpoint-inhibitors.

[182] National Cancer Institute (2022). CAR T Cells: Engineering Patients’ Immune Cells to Treat Their Cancers. https://www.cancer.gov/about-cancer/treatment/research/car-t-cells.

[183] O’Meara M.M., Disis M.L. (2011). Therapeutic Cancer Vaccines and Translating Vaccinomics Science to the Global Health Clinic: Emerging Applications Toward Proof of Concept. OMICS.

[184] Basharat S., Smith A., Darvesh N., Rader T. (2023). Watch List: Top 10 Precision Medicine Technologies and Issues.

[185] Pessoa L.S., Heringer M., Ferrer V.P. (2020). ctDNA as a cancer biomarker: A broad overview. Crit. Rev. Oncol. Hematol..

[186] Pereira Cabral B., da Graça Derengowski Fonseca M., Batista Mota F. (2019). What is the future of cancer care? A technology foresight assessment of experts’ expectations. Econ. Innov. New Technol..

[187] Baxevanis C.N. (2023). Biomarkers in the Era of Precision Oncology. Cancers.

[188] Seyhan A.A., Carini C. (2019). Are innovation and new technologies in precision medicine paving a new era in patients centric care?. J. Transl. Med..

[189] Tsimberidou A.M., Iskander N.G., Hong D.S., Wheler J.J., Falchook G.S., Fu S., Piha-Paul S., Naing M., Janku F., Luthra R. (2012). Personalized Medicine in a Phase I Clinical Trials Program: The MD Anderson Cancer Center Initiative. Clin. Cancer Res..

[190] Radovich M., Kiel P.J., Nance S.M., Niland E.E., Parsley M.E., Ferguson M.E., Jiang G., Ammakkanavar N.R., Einhorn L.H., Cheng L. (2016). Clinical benefit of a precision medicine based approach for guiding treatment of refractory cancers. Oncotarget.

[191] Schwaederle M., Parker B.A., Schwab R.B., Daniels G.A., Piccioni D.E., Kesari S., Helsten T.L., Bazhenova L.A., Romero J., Fanta P.T. (2016). Precision Oncology: The UC San Diego Moores Cancer Center PREDICT Experience. Mol. Cancer. Ther..

[192] Kris M.G., Johnson B.E., Berry L.D., Kwiatkowski D.J., Iafrate A.J., Wistuba I.I., Varella-Garcia M., Franklin W.A., Aronson S.L., Su P. (2014). Using Multiplexed Assays of Oncogenic Drivers in Lung Cancers to Select Targeted Drugs. JAMA.

[193] Stockley T.L., Oza A.M., Berman H.K., Leighl N.B., Knox J.J., Shepherd F.A., Chen E.X., Krzyzanowska M.K., Dhani N., Joshua A.M. (2016). Molecular profiling of advanced solid tumors and patient outcomes with genotype-matched clinical trials: The Princess Margaret IMPACT/COMPACT trial. Genome Med..

[194] Kasztura M., Richard A., Bempong N.E., Loncar D., Flahault A. (2019). Cost-effectiveness of precision medicine: A scoping review. Int. J. Public Health.

[195] Henderson R., Keeling P., French D., Smart D., Sullivan R., Lawler M. (2021). Cost-effectiveness of precision diagnostic testing for precision medicine approaches against non-small-cell lung cancer: A systematic review. Mol. Oncol..

[196] Meehan J., Gray M., Martínez-Pérez C., Kay C., Pang L.Y., Fraser J., Poole A.V., Kunkler L.H., Langdon S.P., Argyle D. (2020). Precision Medicine and the Role of Biomarkers of Radiotherapy Response in Breast Cancer. Front. Oncol..

[197] Mallarkey G., Mangoni A.A. (2015). Targeting precision medicine toxicity: Recent developments. Ther. Adv. Drug Saf..

[198] Sedhom R., Bates-Pappas G.E., Feldman J., Elk R., Gupta A., Fisch M.J., Soto-Perez-de-Celis E. (2024). Tumor Is Not the Only Target: Ensuring Equitable Person-Centered Supportive Care in the Era of Precision Medicine. Am. Soc. Clin. Oncol. Educ. Book.

[199] El-Alti L., Sandman L., Munthe C. (2019). Person Centered Care and Personalized Medicine: Irreconcilable Opposites or Potential Companions?. Health Care Anal..

[200] Sarhadi V.K., Armengol G. (2022). Molecular Biomarkers in Cancer. Biomolecules.

[201] Henry N.L., Hayes D.F. (2012). Cancer biomarkers. Mol. Oncol..

[202] Schmidt K.T., Chau C.H., Price D.K., Figg W.D. (2016). Precision Oncology Medicine: The Clinical Relevance of Patient-Specific Biomarkers Used to Optimize Cancer Treatment. J. Clin. Pharmacol..

[203] National Cancer Institute (2023). Tumor Marker Tests in Common Use. https://www.cancer.gov/about-cancer/diagnosis-staging/diagnosis/tumor-markers-list.

[204] Carmagnani Pestana R., Dias e Silva D., David B.B.L., Schmerling R.A., Filippi R.Z., De Camargo V.P., Mello C.A.L., Donna L.M.G., Munhoz R.R., Varas J.C.C.H. (2024). Sarcoma drug approvals in Latin America compared to the FDA and EMA: An analysis by the LACOG Sarcoma Group. J. Clin. Oncol..

[205] Ivama-Brummell A.M., Marciniuk F.L., Wagner A.K., Osorio-de-Castro C.G.S., Vogler S., Mossialos E., Tavares-de-Andrade C.L., Naci H. (2023). Marketing authorisation and pricing of FDA-approved cancer drugs in Brazil: A retrospective analysis. Lancet Reg. Health Am..

[206] Barrios C.H., Reinert T., Werutsky G. (2019). Access to high-cost drugs for advanced breast cancer in Latin America, particularly trastuzumab. Ecancermedicalscience.

[207] Gomez H.L., Castañeda C., Valencia F., Muñoz-Bermeo R., Torrico Mdel C., Neciosup S. (2020). ABC4 Consensus: First Latin American Meeting—Assessment, Comments, and Application of Its Recommendations. JCO Glob. Oncol..

[208] World Health Organization (2002). National Cancer Control Programmes: Policies and Managerial Guidelines. https://iris.who.int/bitstream/handle/10665/42494/9241545577.pdf?sequence=1.

[209] Pan American Health Organization (2019). Plan of Action for Cervical Cancer Prevention and Control 2018–2030. Washington, D.C. https://iris.paho.org/handle/10665.2/38574.

[210] Pan American Health Organization Breast Cancer: Knowledge Summaries for Health Professionals. https://www.paho.org/en/topics/cancer/breast-cancer-knowledge-summaries-health-professionals.

[211] Vásquez L., Fuentes-Alabi S., Benitez-Majano S., Ribeiro K.B., Abraham M., Agulnik A., Baker J.N., Blanco D.B., Caniza N.A., Cardenas-Aguirre A. (2023). Collaboration for success: The Global Initiative for Childhood Cancer in Latin America. Rev. Panam. Salud Publica.

[212] Ministry of Health of Colombia (2021). MinSalud and Cancerology Institute Will Integrate Public Health and Cancer Control Plans. [Minsalud y Cancerologico Integraran Planes de Salud Public y Control de Cancer]. https://www.minsalud.gov.co/Paginas/Minsalud-y-Cancerologico-integraran-planes-de-salud-publica-y-control-del-cancer.aspx.

[213] Loggetto P., Ritter J., Marx K., Metzger M.L., Lam C.G. (2022). Equity in national cancer control plans in the region of the Americas. Lancet Oncol..

[214] Government Secretariat of Health/National Cancer Institute (2018). Plan Nacional de Control de Cáncer 2018–2022 [National Cancer Control Plan 2018–2022]. https://www.iccp-portal.org/resources/plan-nacional-de-control-de-cancer-2018-2022.

[215] Presidency of the Republic of Brazil (2023). Lei No. 14.758 de 19 de Dezembro de 2023. [Law No. 14,758 of 19 December 2023]. https://www.in.gov.br/en/web/dou/-/lei-n-14.758-de-19-de-dezembro-de-2023-532172581.

[216] Presidency of the Republic of Brazil (2021). Lei No. 14.238, de 19 de Novembro de 2021. https://www.planalto.gov.br/ccivil_03/_Ato2019-2022/2021/Lei/L14238.htm.

[217] Presidency of the Republic of Brazil (2012). Lei No. 12.732, de 22 de Novembro de 2012. https://www.planalto.gov.br/ccivil_03/_ato2011-2014/2012/lei/l12732.htm.

[218] Presidency of Republic of Brazil (2012). Lei No. 12.715 de 17 de Setembro de 2012. [Law No. 12,715 of 17 September 2012]. https://www.planalto.gov.br/ccivil_03/_ato2011-2014/2012/Lei/L12715.htm.

[219] Ministry of Health of Brazil (2021). Strategic Action Plan to Combat Chronic Diseases and Non-Communicable 2021–2030. [Plano de Ações Estratégicas para o Enfrentamento das Doenças Crônicas e Agravos não Transmissíveis no Brasil 2021–2030]. https://www.iccp-portal.org/system/files/plans/NCD%20plan%20Brazil%202021-2030.pdf.

[220] Ministry of Health of Colombia (2012). 10-Year Cancer Control Plan 2012–2021. https://www.minsalud.gov.co/Documents/Plan-Decenal-Cancer/PlanDecenal_ControlCancer_2012-2021.pdf.

[221] Unified Regulatory Information System of Colombia (2010). Ley 1388 de 2010. https://www.suin-juriscol.gov.co/viewDocument.asp?ruta=Leyes/1678530.

[222] Ministry of Health of Colombia (2012). Resolution No. 4496 of 2012. https://www.minsalud.gov.co/sites/rid/Lists/BibliotecaDigital/RIDE/DE/DIJ/Resolucion-4496-de-2012.PDF.

[223] Congress of Colombia (2010). Law 1384 de 2010. https://www.funcionpublica.gov.co/eva/gestornormativo/norma_pdf.php?i=39368.

[224] Ministry of Social Protection (2021). Resolucion No. 0143. https://www.cancer.gov.co/recursos_user/Politicas/Resoluci%C3%B3n_0143_de_2021_(2).pdf.

[225] Ministry of Health of Mexico (2021). Programa de Acción Específico de Prevencion y Control del Cáncer 2021–2024 [Specific Action Program of Prevention and Control of Cancer 2021–2024.]. https://www.gob.mx/cms/uploads/attachment/file/706943/PAE_CAN_cF.pdf.

[226] Ministry of Health of Mexico (2021). Ley General Para la Detección Oportuna del Cáncer en la Infancia y la Adolescencia [General Law for the Timely Detection of Cancer in Childhood and Adolescence]. https://sidofqa.segob.gob.mx/notas/5609564.

[227] Ministry of Health of Panama (2019). Plan Estratégico Nacional para la Prevención y Control del Cáncer 2019–2029 [National Strategic Plan for Cancer Prevention and Control 2019–2029]. https://www.minsa.gob.pa/sites/default/files/publicaciones/plan_estrategico_nacional_para_la_prevencion_y_control_del_cancer_2019_-_2029.pdf.

[228] National Assembly of Panama (2020). Ley 154, de 13 de mayo de 2020 [Law 154, of 13 May 2020]. https://w3.css.gob.pa/wp-content/wdocs/ASAMBLEA%20NACIONAL%20LEY%20154%20DEL%2013%20DE%20MAYO%20DE%202020.pdf.

[229] Ministry of Health of Panama (2008). Decreto Ejecutivo No.382 de 4 de Septiembre de 2008. https://www.gacetaoficial.gob.pa/pdfTemp/26123/13250.pdf.

[230] Ministry of Health of Panama (2022). Resolución No. 291 de 16 de Mayo de 2022. https://www.minsa.gob.pa/sites/default/files/normatividad/resolucion_no_291_de_16_de_mayo_de_2022_normativa.pdf.

[231] Secretariat of Planning and Policies in Science Technology and Innovation (2021). Poblar. https://www.argentina.gob.ar/ciencia/seppcti/poblar.

[232] Ministry of Health of Brazil Genomes Brasil. https://www.gov.br/saude/pt-br/composicao/sectics/decit/genomas-brasil.

[233] Argentinian Government (2018). Mapa de Accionabilidad Genómica Tumoral Argentina (MAGenTa). https://www.argentina.gob.ar/ciencia/dnpe/proyectos/medicina-precision/MAGenTa.

[234] Argentinian Government (2018). Medicina de Precisión en Cáncer y Enfermedades poco Frecuentes (Epof-PAMPA). https://www.argentina.gob.ar/ciencia/dnpe/proyectos/medicina-precision/epof-PAMPA.

[235] Diario Oficial de la Federación (2024). Ley General de Salud [General Health Law] (1984, February 7 rev. 2024, January 3). https://www.diputados.gob.mx/LeyesBiblio/pdf/LGS.pdf.

[236] Legislative Information System (2023). Iniciativa que Adiciona Diversas Disposiciones de la Ley General de Salud, en Materia de Medicina de Precisión, Suscrita por Diputados Integrantes del Grupo Parlamentario del Pan. http://sil.gobernacion.gob.mx/Archivos/Documentos/2023/04/asun_4562318_20230426_1680209383.pdf.

[237] Government of Argentina (2023). Poblar: Un Proyecto federal Para Que la Población Argentina Cuente con un Biobanco Genómico propio. https://www.argentina.gob.ar/noticias/poblar-un-proyecto-federal-para-que-la-poblacion-argentina-cuente-con-un-biobanco-genomico.

[238] Economist Impact (2022). The Journey Towards Health Improvement in Argentina: A Roadmap for Precision Medicine. https://impact.economist.com/perspectives/sites/default/files/economist_impact_precision_medicine_argentina_english_v2.pdf.

[239] Argentinian Government (2017). Biobanco Nacional de Muestras Biológicas: Una Herramienta Esencial Para el Desarrollo de Proyectos de Investigación en el Campo de la Medicina de Precisión. https://www.argentina.gob.ar/ciencia/dnpe/proyectos/medicina-precision/biobanco-nacional.

[240] Argentinian Government (2018). Genómica clínica de Enfermedades Pediátricas-GCEP. https://www.argentina.gob.ar/ciencia/dnpe/proyectos/medicina-precision/enfermedades-pediatricas.

[241] Centro de Estudios Regulatorios (2023). Dapre, Ley 2287 de 2023. https://www.cerlatam.com/normatividad/dapre-ley-2287-de-2023/.

[242] Yan J.T., Jin Y., Lo E., Chen Y., Hanlon Newell A.E., Kong Y., Inge L.J. (2023). Real-World Biomarker Test Utilization and Subsequent Treatment in Patients with Early-Stage Non-small Cell Lung Cancer in the United States, 2011−2021. Oncol. Ther..

[243] Wu N., Ge W., Quek R.G., Gleeson M., Pouliot J.F., Dietz H., Jalbert J.J., Harnett J., Antonia S.J. (2022). Trends in Real-World Biomarker Testing and Overall Survival in US Patients with Advanced Non-Small-Cell Lung Cancer. Future Oncol..

[244] Bruno D.S., Hess L.M., Li X., Su E.W., Patel M. (2022). Disparities in Biomarker Testing and Clinical Trial Enrollment Among Patients With Lung, Breast, or Colorectal Cancers in the United States. JCO Precis. Oncol..

[245] Dieguez G., Carioto J. (2022). The Landscape of Biomarker Testing Coverage in the United States. https://www.fightcancer.org/sites/default/files/the_landscape_of_biomarker_testing_coverage_in_the_u_s_0.pdf.

[246] Norris R.P., Dew R., Sharp L., Greystoke A., Rice S., Johnell K., Todd A. (2020). Are there socio-economic inequalities in utilization of predictive biomarker tests and biological and precision therapies for cancer? A systematic review and meta-analysis. BMC Med..

[247] Normanno N., Apostolidis K., Wolf A., Al Dieri R., Deans Z., Fairley J. (2022). Access and quality of biomarker testing for precision oncology in Europe. Eur. J. Cancer.

[248] EFPIA (2021). IQNPath. European Cancer Patient Coallition. Unlocking the Potential of Precision Medicine in Europe—Improving Cancer Care Through Broader Access to Quality Biomarker Testing. https://www.efpia.eu/media/589673/biomarker-testing-summary-final-version.pdf.

[249] Memorial Sloan Kettering Cancer Center Library Precision Medicine: U.S (2024). Gov’t Initiatives. https://libguides.mskcc.org/precisionmedicine/usgovt.

[250] National Cancer Institute (2023). NCI-supported Precision Medicine Oncology Research Activities. https://dctd.cancer.gov/MajorInitiatives/NCI-supported_activities_in_precision_medicine.htm.

[251] National Cancer Institute (2024). Center for Cancer Genomics. Supporting Genomic Science to Improve Cancer Diagnosis, Treatments, and Outcomes. https://www.cancer.gov/ccg/.

[252] National Institutes of Health (2024). All of Us Research Program. https://allofus.nih.gov/.

[253] National Institutes of Health (2024). Transdisciplinary Collaborative Centers for Health Disparities Research Program. https://www.nimhd.nih.gov/node/22006.

[254] Beccia F., Hoxhaj I., Castagna C., Strohäker T., Cadeddu C., Ricciardi W., Boccia S. (2022). An overview of Personalized Medicine landscape and policies in the European Union. Eur. J. Public Health.

[255] Phanthunane C., Pongcharoen S., Pannarunothai S., Roboon J., Phanthunane P., Nontarak J. (2024). Precision medicine in Asia enhanced by next-generation sequencing: Implications for Thailand through a scoping review and interview study. Clin. Transl. Sci..

[256] Wong E., Bertin N., Hebrard M., Tirado-Magallanes R., Bellis C., Lim W.K., Chua C.Y., Tong P.M.L., Chua R., Mak K. (2023). The Singapore National Precision Medicine Strategy. Nat. Genet..

[257] Susanti S., Subhandi Bakhtiar H., Prasetyo H. (2023). Protection of Genetic Data in Health Services Based on Genomics Technology in Indonesia. Asian J. Healthy Sci..

[258] Food and Drugs Administration (1998). Herceptin. https://www.accessdata.fda.gov/drugsatfda_docs/label/2010/103792s5250lbl.pdf.

[259] European Medicines Agency Herceptin. https://www.ema.europa.eu/en/medicines/human/EPAR/herceptin.

[260] Government of Mexico Consulta de Registros Sanitarios. https://tramiteselectronicos02.cofepris.gob.mx/BuscadorPublicoRegistrosSanitarios/BusquedaRegistroSanitario.aspx.

[261] National Human Genome Research Institute (2024). NHGRI History and Timeline of Events. https://www.genome.gov/about-nhgri/Brief-History-Timeline.

[262] Agencia Nacional de Vigilancia Sanitaria Herceptine. https://consultas.anvisa.gov.br/#/medicamentos/8090?substancia=23119.

[263] Food and Drugs Administration (2001). Gleevec. https://www.accessdata.fda.gov/drugsatfda_docs/label/2008/021588s024lbl.pdf.

[264] European Medicines Agency Glivec. https://www.ema.europa.eu/en/medicines/human/EPAR/glivec.

[265] Agencia Nacional de Vigilancia Sanitaria Glivec. https://consultas.anvisa.gov.br/#/medicamentos/5757?numeroRegistro=100680174.

[266] Collins F.S., Hamburg M.A. (2013). First FDA Authorization for Next-Generation Sequencer. N. Engl. J. Med..

[267] European Medicines Agency Keytruda. https://www.ema.europa.eu/en/medicines/human/EPAR/keytruda.

[268] Food and Drugs Administration (2014). Food and Drugs Administration. https://www.accessdata.fda.gov/drugsatfda_docs/label/2020/125514s066lbl.pdf.

[269] International Consortium for Personalized Medicine (2023). ICPerMed. https://www.icpermed.eu/.

[270] European Commission Personalised Medicine. https://research-and-innovation.ec.europa.eu/research-area/health/personalised-medicine_en.

[271] Ministry of Human Rights and Citizenship (2020). Governo Federal Lança Programa Inédito de Medicina de Precisão. https://www.gov.br/mdh/pt-br/assuntos/noticias/2020-2/outubro/governo-federal-lanca-programa-inedito-de-medicina-de-precisao.

[272] Agencia Nacional de Vigilancia Sanitaria Registrados Medicamentos Inovadores para Câncer de Pele. https://antigo.anvisa.gov.br/resultado-de-busca?p_p_id=101&p_p_lifecycle=0&p_p_state=maximized&p_p_mode=view&p_p_col_id=column-1&p_p_col_count=1&_101_struts_action=%2Fasset_publisher%2Fview_content&_101_assetEntryId=3037244&_101_type=content&_101_groupId=219201&_101_urlTitle=registrados-medicamentos-inovadores-para-cancer-de-pele&inheritRedirect=true.

[273] Genomics England 100,000 Genomes Project. https://www.genomicsengland.co.uk/initiatives/100000-genomes-project.

[274] European Commission (2024). European “1+ Million Genomes” Initiative. https://digital-strategy.ec.europa.eu/en/policies/1-million-genomes.

[275] Chong H.Y., Allotey P.A., Chaiyakunapruk N. (2018). Current landscape of personalized medicine adoption and implementation in Southeast Asia. BMC Med. Genom..

[276] Chumnumwat S., Lu Z.H., Sukasem C., Winther M.D., Capule F.R., Abdul Hamid A.A., Bhandari B., Chaikledkaew U., Chanhom N., Chantarangsu S. (2019). Southeast Asian Pharmacogenomics Research Network (SEAPharm): Current Status and Perspectives. Public Health Genom..

[277] de Vries J., Tindana P., Littler K., Ramsay M., Rotimi C., Abayomi A., Mulder N., Mayosi B.M. (2015). The H3Africa policy framework: Negotiating fairness in genomics. Trends Genet..

[278] Mulder N., Abimiku A., Adebamowo S.N., de Vries J., Matimba A., Olowoyo P., Ramsey M., Skelton M., Stein D.J. (2018). H3Africa: Current perspectives. Pharmgenomics Pers. Med..

[279] NHS England NHS Genomic Medicine Service. https://www.england.nhs.uk/genomics/nhs-genomic-med-service/.

[280] Neuhaus C.P., Pacia D.M., Crane J.T., Maschke K.J., Berlinger N. (2023). All of Us and the Promise of Precision Medicine: Achieving Equitable Access for Federally Qualified Health Center Patients. J. Pers. Med..

[281] Williams J.S., Walker R.J., Egede L.E. (2016). Achieving Equity in an Evolving Healthcare System: Opportunities and Challenges. Am. J. Med. Sci..

[282] Ory M.G., Adepoju O.E., Ramos K.S., Silva P.S., Vollmer Dahlke D. (2023). Health equity innovation in precision medicine: Current challenges and future directions. Front. Public Health.

[283] Botham J., Shilling V., Jones J. (2021). Patient and public understanding of the concept of ‘personalised medicine’ in relation to cancer treatment: A systematic review. Future Health J..

[284] Yelne S., Chaudhary M., Dod K., Sayyad A., Sharma R. (2023). Harnessing the Power of AI: A Comprehensive Review of Its Impact and Challenges in Nursing Science and Healthcare. Cureus.

[285] Corti C., Cobanaj M., Dee E.C., Criscitiello C., Tolaney S.M., Celi L.A., Curigliano G. (2023). Artificial intelligence in cancer research and precision medicine: Applications, limitations and priorities to drive transformation in the delivery of equitable and unbiased care. Cancer Treat. Rev..

[286] Seetharam K., Kagiyama N., Sengupta P.P. (2019). Application of mobile health, telemedicine and artificial intelligence to echocardiography. Echo. Res. Pract..

[287] Goss P.E., Lee B.L., Badovinac-Crnjevic T., Strasser-Weippl K., Chavarri-Guerra Y., Louis J.S., Villarreal-Garza C., Unger-Saldaña K., Ferreyra M., Debiasi M. (2013). Planning cancer control in Latin America and the Caribbean. Lancet Oncol..

[288] Ferreira C.G., Achatz M.I., Ashton-Prolla P., Begnami M.D., Marchini F.K., Stefani S.D. (2016). Brazilian health-care policy for targeted oncology therapies and companion diagnostic testing. Lancet Oncol..

[289] Fong H., Harris E. (2015). Technology, innovation and health equity. Bull. World Health Organ..

[290] Gallifant J., Nakayama L.F., Gichoya J.W., Pierce R., Celi L.A. (2023). Equity should be fundamental to the emergence of innovation. PLoS Digit. Health.

[291] Temporão J.G., Santini L.A., Santos ATCdos Fernandes F.M.B., Zoss W.P. (2022). Current and future challenges of the use of precision medicine in cancer diagnosis and treatment in Brazil. Cad. Saude Publica..

[292] Shirdarreh M., Aziza O., Pezo R.C., Jerzak K.J., Warner E. (2021). Patients’ and Oncologists’ Knowledge and Expectations Regarding Tumor Multigene Next-Generation Sequencing: A Narrative Review. Oncologist.

[293] Dittrich R. Healthcare Priority Setting in the Courts: A Reflection on Decision-Making When Healthcare Priority Setting Is Brought to Court. https://f1000research.com/documents/6-242.

[294] Robles M.Y. (2016). El derecho a la salud en la jurisprudencia de la corte Interamericana De Derechos Humanos (2004–2014). Cuest. Constitucl..

[295] Raez L.E., Santos E.S., Rolfo C., Lopes G., Barrios C., Cardona A., Mas L.A., Arrieta O., Richardet E., Vallejos C. (2017). Challenges in Facing the Lung Cancer Epidemic and Treating Advanced Disease in Latin America. Clin. Lung Cancer.

[296] Raez L.E., Cardona A.F., Santos E.S., Catoe H., Rolfo C., Lopes G., Barrios C., Mas L.A., Vallejos C., Zatarain-Barrón Z.L. (2018). The burden of lung cancer in Latin-America and challenges in the access to genomic profiling, immunotherapy and targeted treatments. Lung Cancer.

[297] Balogun O.D., Olopade O.I. (2021). Addressing health disparities in cancer with genomics. Nat. Rev. Genet..

[298] Ginsburg G.S., Phillips K.A. (2018). Precision Medicine: From Science To Value. Health Aff..

[299] Bertier G., Carrot-Zhang J., Ragoussis V., Joly Y. (2016). Integrating precision cancer medicine into healthcare—Policy, practice, and research challenges. Genome Med..

[300] Lotsberg M.L., D’mello Peters S.A. (2022). Publication Bias in Precision Oncology and Cancer Biomarker Research. Challenges and Possible Implications.

[301] Zavala V.A., Serrano-Gomez S.J., Dutil J., Fejerman L. (2019). Genetic Epidemiology of Breast Cancer in Latin America. Genes.

[302] Popejoy A.B., Fullerton S.M. (2016). Genomics is failing on diversity. Nature.

[303] Vargas R.J., Cobar O.M. (2021). The Urgent Need for Management of Biological Samples and Data Accessibility in Latin America. Front. Pharmacol..

[304] Cooke Bailey J.N., Bush W.S., Crawford D.C. (2020). Editorial: The Importance of Diversity in Precision Medicine Research. Front. Genet..

[305] Gössling G., Rebelatto T.F., Villarreal-Garza C., Ferrigno A.S., Bretel D., Sala R., Giacomazzi J., William W.N., Werutsky G. (2023). Current Scenario of Clinical Cancer Research in Latin America and the Caribbean. Curr. Oncol..

[306] Edsjö A., Holmquist L., Geoerger B., Nowak F., Gomon G., Alix-Panabières C., Ploeger C., Lassen U., Le Tourneau C., Lehtiö J. (2023). Precision cancer medicine: Concepts, current practice, and future developments. J. Intern. Med..

[307] Al Meslamani A.Z. (2023). The future of precision medicine in oncology. Expert. Rev. Precis. Med. Drug Dev..

[308] Bestari M.B., Joewono I.R., Syam A.F. (2024). A Quest for Survival: A Review of the Early Biomarkers of Pancreatic Cancer and the Most Effective Approaches at Present. Biomolecules.

[309] Lopez-Gonzalez L., Sanchez Cendra A., Sanchez Cendra C., Roberts Cervantes E.D., Espinosa J.C., Pekarek T., Fraile-Martinez O., García-Montero C., Rodriguez-Sloker A.M. (2024). Exploring Biomarkers in Breast Cancer: Hallmarks of Diagnosis, Treatment, and Follow-Up in Clinical Practice. Medicina.

[310] Amur S., LaVange L., Zineh I., Buckman-Garner S., Woodcock J. (2015). Biomarker Qualification: Toward a Multiple Stakeholder Framework for Biomarker Development, Regulatory Acceptance, and Utilization. Clin. Pharmacol. Ther..

[311] Hayes D.F. (2015). Biomarker validation and testing. Mol. Oncol..

[312] Burke W. (2014). Genetic Tests: Clinical Validity and Clinical Utility. Curr. Protoc. Hum. Genet..

[313] National Cancer Institute (2021). Biomarker Testing for Cancer Treatment. https://www.cancer.gov/about-cancer/treatment/types/biomarker-testing-cancer-treatment.

[314] West H.J., Lovly C.M. (2023). Ferrying Oncologists Across the Chasm of Interpreting Biomarker Testing Reports: Systematic Support Needed to Improve Care and Decrease Disparities. JCO Oncol. Pract..

[315] Moore D.C., Guinigundo A.S. (2023). The Advanced Practitioner’s Role in the Rapidly Evolving Landscape of Precision Medicine. J. Adv. Pract. Oncol..

[316] Al-Dewik N.I., Younes S.N., Essa M.M., Pathak S., Qoronfleh M.W. (2022). Making Biomarkers Relevant to Healthcare Innovation and Precision Medicine. Processes.

[317] Bakker E., Starokozhko V., Kraaijvanger J.W.M., Heerspink H.J.L., Mol P.G.M. (2023). Precision medicine in regulatory decision making: Biomarkers used for patient selection in European Public Assessment Reports from 2018 to 2020. Clin. Transl. Sci..

[318] Tseng T.S. (2016). Impact of social behavioral and genomic sciences on translational cancer research. Transl. Cancer Res..

[319] Alvarez-Gomez R.M., De la Fuente-Hernandez M.A., Herrera-Montalvo L., Hidalgo-Miranda A. (2021). Challenges of diagnostic genomics in Latin America. Curr. Opin. Genet. Dev..

[320] Moye-Holz D., Vogler S. (2022). Comparison of Prices and Affordability of Cancer Medicines in 16 Countries in Europe and Latin America. Appl. Health Econ. Health Policy.

[321] Pichon-Riviere A., Garay O.U., Augustovski F., Vallejos C., Huayanay L., Bueno M.P.N., Rodriguez A., Andrade C.J.C., Buendía J.A., Drummond M. (2015). Implications of global pricing policies on access to innovative drugs: The case of trastuzumab in seven Latin American countries. Int. J. Technol. Assess. Health Care.

[322] UNESCO (2020). Fact Sheet No. 59-Global Investments in R&D. https://uis.unesco.org/sites/default/files/documents/fs59-global-investments-rd-2020-en.pdf.

[323] The Lancet Oncology (2018). Violence, crime, corruption, and cuts in public spending: How to re-establish cancer control in Latin America?. Lancet Oncol..

[324] FIFARMA & IQVIA (2024). Patient WAIT Indicator 2023—Latin America: Final Report. https://fifarma.org/wp-content/uploads/2024/03/IQVIA-FIFARMA_WAIT-Indicator-2023_FinalPresentation_5Mar24_vS.pdf.

[325] Gilardino R.E., Mejía A., Guarín D., Rey-Ares L., Perez A. (2020). Implementing Health Technology Assessments in Latin America: Looking at the Past, Mirroring the Future. A Perspective from the ISPOR Health Technology Assessment Roundtable in Latin America. Value Health Reg. Issues.

[326] Lessa F., Caccavo F., Curtis S., Ouimet-Rathé S., Lemgruber A. (2017). Strengthening and implementing health technology assessment and the decision-making process in the Region of the Americas. Rev. Panam. Salud. Pública.

[327] World Health Organization (2024). Guidance for Human Genome Data Collection, Access, Use and Sharing.

